# An Overview of Macrolide Resistance in Streptococci: Prevalence, Mobile Elements and Dynamics

**DOI:** 10.3390/microorganisms10122316

**Published:** 2022-11-23

**Authors:** Dàmaris Berbel, Aida González-Díaz, Guillem López de Egea, Jordi Càmara, Carmen Ardanuy

**Affiliations:** 1 Microbiology Department, Hospital Universitari de Bellvitge, IDIBELL-UB, 08907 Barcelona, Spain; 2 Research Network for Respiratory Diseases (CIBERES), ISCIII, 28020 Madrid, Spain; 3 Department of Pathology and Experimental Therapeutics, School of Medicine, University of Barcelona, 08007 Barcelona, Spain

**Keywords:** macrolide resistance, *Streptococcus*, *Streptococcus pneumoniae*, *Streptococcus pyogenes*, GAS, pneumococcus, mobile genetic elements, ICE, IME

## Abstract

Streptococcal infections are usually treated with beta-lactam antibiotics, but, in case of allergic patients or reduced antibiotic susceptibility, macrolides and fluoroquinolones are the main alternatives. This work focuses on studying macrolide resistance rates, genetic associated determinants and antibiotic consumption data in Spain, Europe and also on a global scale. Macrolide resistance (MR) determinants, such as ribosomal methylases (*erm*(B), *erm*(TR), *erm*(T)) or active antibiotic efflux pumps and ribosomal protectors (*mef*(A/E)-*mrs*(D)), are differently distributed worldwide and associated with different clonal lineages and mobile genetic elements. MR rates vary together depending on clonal dynamics and on antibiotic consumption applying selective pressure. Among *Streptococcus*, higher MR rates are found in the viridans group, *Streptococcus pneumoniae* and *Streptococcus agalactiae*, and lower MR rates are described in *Streptococcus pyogenes*. When considering different geographic areas, higher resistance rates are usually found in East-Asian countries and milder or lower in the US and Europe. Unfortunately, the availability of data varies also between countries; it is scarce in low- and middle- income countries from Africa and South America. Thus, surveillance studies of macrolide resistance rates and the resistance determinants involved should be promoted to complete global knowledge among macrolide resistance dynamics.

## 1. Streptococcus

The genus *Streptococcus* comprises a large number of species found in humans and animals as part of the normal microbiota. Some of them had successfully adapted to progress from colonization to cause invasive disease, involving different bacterial processes, such as adherence and invasion or resistance to host immune responses [1,2]. While some streptococcal species are almost exclusively restricted to a specific host, such as *Streptococcus suis* in pigs, others can be found in multiple hosts, such as *Streptococcus agalactiae*. Moreover, these streptococci that are not usually part of the human microbiota, such as *Streptococcus suis,* can cause zoonotic infections in the process of animal and human interaction. Then, antibiotic animal policies to treat streptococcal infections may have a direct impact on streptococci causing human infections [1,2].

As human pathogens, streptococci can cause a wide variety of infections, such as pneumonia, meningitis, endocarditis, otitis media, sepsis or skin and soft-tissue infections. Among them, *Streptococcus pneumoniae* and *Streptococcus pyogenes* are major human pathogens; they are an important cause of morbidity and mortality worldwide and are the main representative of the mitis and pyogenic group of streptococci [1,2,3,4,5].

Although streptococcal infections are mainly treated with beta-lactam antibiotics, macrolides are an important group of antibiotics used either as combined therapy in severe infections, such as community-acquired pneumonia, as an alternative therapy in beta-lactam allergic patients or as adjunctive therapy for its protein inhibition effect in toxin-related diseases, such as streptococcal toxic shock syndrome (SSTS). This review provides a synopsis of macrolide resistance in pathogenic *Streptococcus* species regarding rates (Table 1 and Table S1) and mechanisms of resistance (Table 2), and also the major drivers of resistance: mobile genetic elements (MGE), clonal dynamics and antibiotic consumption [1,2,6,7]. Worldwide macrolide resistance rates are summarized in Table 1 and more extendedly studied in Table S1.

## 2. Macrolides and Resistance Mechanisms

Macrolides are a group of antibiotics made up of a macrolactone ring, varying in size depending on the carbon atom composition of the lactone ring, with neutral or amino sugar groups attached [6,7]. Macrolides classification is based on the carbon atom composition of the lactone ring: 14-member ring, erythromycin and clarithromycin; 15-member ring, azithromycin; and 16-member ring, spiramycin and josamycin. Erythromycin, naturally produced by *Streptomyces erythreus,* was discovered in 1952. With the exception of spiramycin (produced by *Streptomyces ambofaciens*), the remaining macrolides are semisynthetic, as a result of subsequent modifications to improve the pharmacokinetics and spectrum of activity. Macrolides inhibit peptide chain elongation by reversibly binding to 23S rRNA at the peptidyl-tRNA binding region, blocking the nascent peptide channel exit, inhibiting translocation, and thus peptidyl-tRNA drop off leading to abortion of translation. Smaller macrolides (14-and 15-membered) partially block the channel in a manner that allows only short oligopeptides (6–8 Aa) to form, but 16-membered macrolides fully block the channel and cause ribosomal disassociation [6,7,8]. The emergence of macrolide resistance is a cause for concern and macrolides have been included in the list of critically important antimicrobials for Human Medicine of the World Health Organization (WHO) [9].

Resistance to macrolides in the genus *Streptococcus* is due to three different mechanisms: ribosomal post- and pre-transcriptional modifications, active expulsion of the antibiotic by efflux pumps and target protection [6,8,10,11,12,13]. These resistance mechanisms usually confer resistance to other antibiotic groups which have their target site in the 50S ribosomal unit, such as lincosamides or streptogramins. This fact is the basis of phenotypic classification of macrolide and lincosamide resistance.

The post-transcriptional ribosomal modification is performed by methylases encoded by the *erm* (erythromycin ribosome methylase) genes that mono- or dimethylate the A2058 in the V domain of 23S rRNA [6,10,11]. This methylation causes a conformational change in the ribosomal peptidyl transferase center (PTC) 50S ribosomal subunit, and thus high-level resistance to macrolides, lincosamides and group B streptogramins (MLS_B_ phenotype) that can be constitutively (cMLS_B_) or inducibly (iMLS_B_) expressed [6,10,11]. When inducible, clindamycin may appear susceptible, but, in the presence of a macrolide, resistance rises; for this reason, antibiotic susceptibility testing should include induction tests, such as a screening by disk–diffusion D-zone to avoid false susceptibility results [11]. More than 20 classes of *erm* genes have been identified [7], but in streptococci the most frequently found are *erm*(B), *erm*(TR) (also known as a subclass of *erm*(A)) and *erm*(T) [6,10,11,14,15,16,17,18]. 

Resistance due to ribosomal mutations in the 23S rRNA or L4 and L22 proteins is very rare in streptococci, conferring different resistance phenotypes depending on the mutations found [6]. For instance, mutations in 23S rRNA, or near the macrolide binding residue A2058, result in different levels of macrolide resistance, depending on the copies of the rRNA operons mutated, which means that high-level resistance requires mutation of most of these operons [6,19,20,21]. Regarding riboproteins, substitutions in the L4 protein (*rplD*) such as K63E, deletions in L4, such as Del65WR66 or deletions of the 82ME84 in the L22 (*rplV*) are the most frequently found associated with macrolide resistance [6,20,22,23,24]. 

The active expulsion of the antibiotic is determined by the major facilitator superfamily (MFS) efflux pumps encoded in the *mef* gene (macrolide efflux family) [6,10,11,17,18,22]. This mechanism confers low-level resistance to 14- and 15-membered ring macrolides (erythromycin and azithromycin), while there is no resistance to 16-membered macrolides, lincosamides, and streptogramins producing the M-phenotype [6,10,13]. Several allelic variants or subclasses of the *mef* gene have been described, although they share 90% identity between them: *mef*(A) originally reported in *S. pyogenes*, *mef*(E) later identified in *S. pneumoniae*, *mef*(I) also in pneumococcus, and *mef*(O) in Group A *Streptococcus* (GAS) [6,10,11,14,22,25,26]. The *mef* gene was originally identified as the resistance determinant responsible for type M resistance to macrolides, but *mrs*(D) (formerly known as *mel*) always adjacent to *mef*(A) and *mef*(E) has been recently related to macrolide resistance [27,28,29,30,31]. In this way, the Mef protein is responsible for the expulsion of the antibiotic, while [12,27] Mrs(D), as an ABC-F protein, is involved in antibiotic resistance through target protection by driving dissociation of bound antibiotic molecules from the ribosome, safeguarding ribosomal function [32]. 

The worldwide distribution of the macrolide resistance determinants may vary associated to a specific clonal distribution or a horizontal transmission by mobile genetic elements. Table 2 summarizes the frequency of the macrolide resistance determinants (*erm*(B), *erm*(TR), *erm*(T) and *mef*(A/E)) among different geographical regions.

## 3. Macrolide Resistance in β-Haemolitic *Streptococcus*

**Figure 1:**
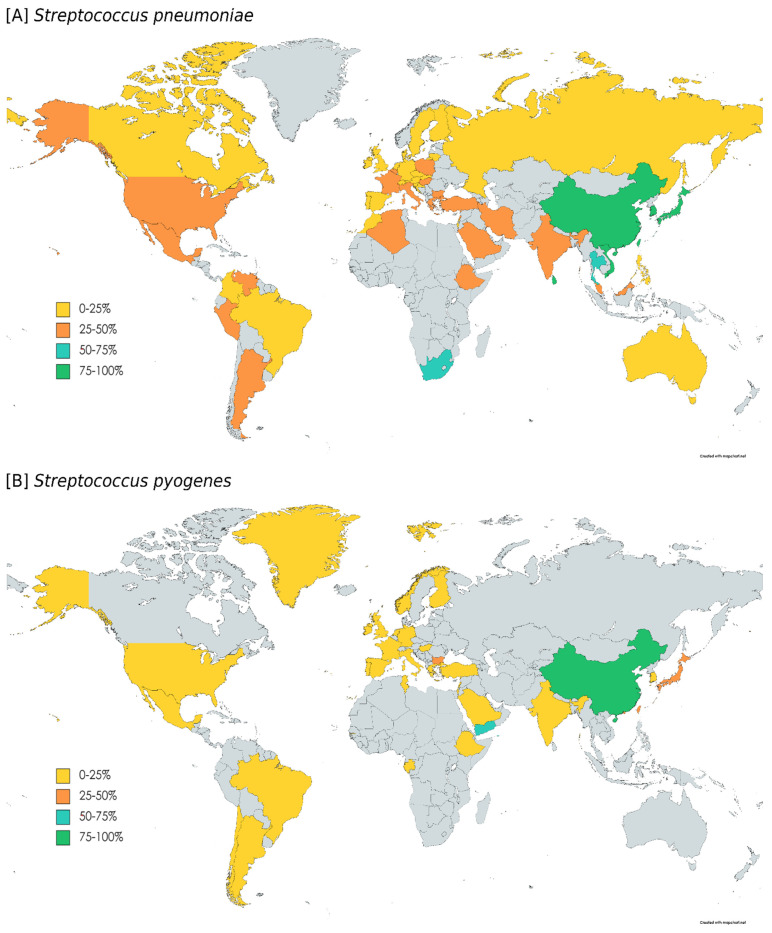
Macrolide resistance rates in the world: *Streptococcus pneumoniae* (**A**) and *Streptococcus pyogenes* (**B**). Macrolide resistance rates reported in Supplementary Table S1 are depicted as yellow (0–25%), orange (25–50%), blue (50–75%) and green (75–100%) zones, and grey zones mean countries with no data.

The first macrolide-resistant *S. pyogenes* (MR-GAS) was reported in 1968 and, since then, the macrolide resistance rates have varied geographically and temporally, associated with clonal dynamics, outbreaks and antibiotic consumption [33]. In *S. pyogenes*, macrolide resistance rates vary widely depending on geographic areas (Table 1); rates tend to be very low (<4%) in some European countries, mild (5–39%) in other European countries and the United States of America (USA), and higher (>40%) in Asian countries (Figure 1). Unfortunately, epidemiological studies of macrolide-resistant (MR) GAS in Africa and South America are scarce.

In Spain, macrolide-resistant *S. pyogenes* emerged in the early 1990s and the M phenotype became predominant by clones encoding *mef*(A) [17,34,35]. The MR-GAS prevalence rapidly rose, reaching a peak from 26% to 44% during the early 2000s in both invasive and non-invasive isolates. This increase was related to a high prevalence of clones associated with *erm*(B) genes and the MLS_B_ phenotype, such as *emm*11-ST403 or *emm*28-ST52. Geographical variations were also related to outbreaks, as occurred in Barcelona among the intravenous drug use population [17,34,35]. Later, most of the epidemiological studies have shown a reduction in macrolide resistance with rates below 10% after 2005, linked to a decline in prevalence of major MR clones [3,17,36]. The decreasing trend continued in time, with current MR rates in the range of 4% in Northern Spain in 2015, 8.5% in a national surveillance study in 2019, or 11.7% in 2016–2018 in adults from Barcelona (Northeast) [37,38]. A similar situation was described in Portugal with a peak of resistance of around 21% in the early 2000s, associated with both M and MLS_B_ phenotypes (Table 2), followed by a decrease of MR rates to 4.4% in 2013, which has been maintained to date with a progressive disappearance of the M phenotype [16,39,40]. Similar data were found in other Southern European countries, such as Greece with resistance rates from 15 to 25% (2003–2017) or 18% in Italy (2000–2013) [41,42,43,44,45]. Data from Northern European countries, such as Norway, Finland, Germany, The Netherlands, United Kingdom (UK) and Ireland showed macrolide resistance rates below 5% in different periods [46,47,48,49,50]. In Europe, the major clones encoding *mef*(A) were *emm*4-ST39 and *emm*75-ST49 and, after the 2000s, mostly *emm*12-ST36. The *erm*(B) was associated with *emm*28-ST52 at first and later it was found in *emm*11-ST403 lineages, and *erm*(TR) raised recently together with *emm*77-ST63 [3,16,17,34,35,36,39,40,41,42,43,44,45,46,47,49,50,51].

**Table 1:** Global distribution of macrolide resistance rates summarized by streptococcal species. This table synthesizes what is described in Supplementary Table S1, where the references can be consulted.

		*Streptococcus pneumoniae*			*Streptococcus pyogenes*			*Streptococcus dysgalactiae*			*Streptococcus agalactiae*		
Continent	Region	Period	Mean (%)	Range (%)	Period	Mean (%)	Range (%)	Period	Mean (%)	Range (%)	Period	Mean (%)	Range (%)
Africa	Eastern	2016–2017	33.6	*	2021–2021	6.1	*	-	-	-	2021–2021	20.7	*
	Middle	-	-	-	2012–2013	11.0	*	-	-	-	-	-	-
	Northern	1998–2014	15.6	9.4–31.0	2000–2013	4.6	3.6–5.2	-	-	-	-	-	-
	Western	-	-	-	2004–2012	11.1	*	2012–2013	28.1	*	2018–2018	30.3	*
America	Central	2003–2004	29.0	*	1999–2009	4.6	*	-	-	-	-	-	-
	Northern	1986–2019	23.2	3.5–47.3	1997–2019	16.5	3.9–25.0	1997–2004	19.3	-	1997–2004	52.0	26.6–57.0
	South	2003–2019	26.3	0–85.9	1996–2012	4.0	2.4–15.4	1979–2013	16.5	13.9–26.1	2014–2018	18.6	17.3–25.0
Asia	Eastern	2000–2020	89,6	56.6–100	1998–2019	40.2	1.2–95.0	1993–2016	24.3	10.3–71.4	2003–2021	35.5	29.1–40.7
	Southeastern	2008–2009	49.9	5.3–82.9	-	-	-	-	-	-	-	-	-
	Southern	2008–2013	48.2	17.4–79.0	1986–2017	9.4	2.9–53.0	-	-	-	-	-	-
	Western	2003–2016	42.4	14.0–67.7	1996–2019	8.5	4.3–61.3	1995–2011	12,4	6.0–38.9	-	-	-
Europe	Eastern	1995–2016	25.2	4.1–49.1	2013–2017	13.0	10.5–31.0	2008–2017	21.4	*	2016–2018	27.4	22.0–30.4
	Northern	1996–2018	5.7	0.3–24.2	1993–2016	9.8	1.6–11.9	2000–2006	7.3	5.4–15.1	2000–2018	12.3	3.4–17.5
	Southern	1997–2019	21.4	6.1–49.4	1993–2019	14.8	3.2–46.8	2000–2015	23.2	16.7–31.3	2001–2020	33.4	15.7–26.0
	Western	1992–2016	21.3	2.9–53.1	2003–2013	3.4	1.4–4.0	2005–2010	13.2	5.1–26.4	2005–2019	27.1	5.3–34.1
Oceania	Australia	1999–2017	20.4	13.0–31.0	-	-	-	-	-	-	2016–2019	32.0	*

- No data. * Single study.

In the USA, a study showed 9% of MR in the 1990s was mostly due to isolates with the M phenotype [52]. After that, the MLS_B_ phenotype became predominant and the rate of MR increased to 15% in 2015 [53,54]. More recently, the spread of different epidemic strains changed the epidemiology of MR lineages in the USA: the dominant lineage until 2016 (*emm*11-ST403/*erm*(B)) was replaced by *emm*92-ST82, leading to an increase of MR up to 19% in 2017 [55,56,57]. This was related to the spread of a plasmid containing *erm*(T) among successful lineages such as *emm*92-ST82, *emm*4-ST39 and *emm*77-ST399, which changed the epidemiology of the genetic elements associated with MR (Table 2) [55,56,57]. Lately the spread of an *emm*49-ST433/*erm*(TR) clone, balanced the proportion of *erm*(TR)/*erm*(T) and increased MR rates up to 25% in 2019 [57]. Different studies from South America between 1996 and 2009 showed MR rates below 5% in Chile and Argentina 1996–2009 [58,59,60]. Nevertheless, a study from Brazil reported higher resistance rates (15.4%) from 2008 to 2012 [61]. 

Asian countries have the highest rates of MR worldwide; in China, MR increased from 15% in 2000 to 95% in 2016 mainly associated with the spread of *emm*12-ST36/*erm*(B) and *emm*1-ST28/*erm*(B), which accounted for 80% of MR GAS (Table 2) [62,63]. In Taiwan, MR rates also increased from 18% in 2000–2009 to 58% in 2010–2019 linked to the *emm*12-ST36/*erm*(B) lineage [64]. Similar findings were described in Japan with 40% MR rates in different studies from 2011–2018, but with a higher prevalence of the M phenotype (Table 2) [65,66]. Contrarily, in South Korea, very low resistance rates (<5%) from 2003–2019 were reported [67,68]. In Africa and Middle East countries, the reported resistance rates are below 10%, except for Yemen with 61.3% of MR in 2012 [69,70,71,72].

Other β-haemolitic streptococci as *Streptococcus agalactiae* and *Streptococcus dysgalactiae* subsp *equisimilis* (SDSE) have higher macrolide resistance rates worldwide (Table 1). Probably their ability to colonize and cause disease in humans and animals together with the high antibiotic use in the latter have contributed to these rates [73]. MR rates among SDSE from Europe and the USA were about 19% between 1997 and 2006, but higher MR rates have been described in later periods, such as 26.4% in France (2006–2010), 31% in Italy (2000–2010) and 21.4% in Southern Hungary (2017–2018) [52,74,75,76,77]. Asian countries showed the highest MR rates, as much as 71.4% in China (2007–2015), 42% in Korea (2012–2016) and 45.4% in Southern Taiwan (1993–2010), but milder in Central Taiwan (24% in 2007–2011) and Japan (18.4% in 2010–2013) [78,79,80,81,82]. Little studies report MR in Africa and the Middle East; there is a report from Gabon of 28.1% MR rate in 2013 [83] and 8.6% MR in Israel in the 2007–2011 period [84]. Overall, the MLS_B_ phenotype is the most frequently described among SDSE associated with both, *erm*(B) and *erm*(TR) (Table 2). Nevertheless, some exceptions could be found, such as in Taiwan when *mef*(A) accounted for more than a half of MR SDSE isolates [80,81]. Moreover, different lineages are associated with macrolide resistance in SDSE without a specific lineage trend [76,79,81,82,85]. 

Worldwide, macrolide resistant rates are also high in *S. agalactiae*. As occurred with other streptococci, Asian countries have the highest resistance prevalence, up to 70% in China in 2015 and 36% in Korea between 2018 and 2021 [86,87]. Nevertheless, high resistance rates were reported in the USA (55% of MR in the last decade) and some European countries with rates ranging from 17.5% in Denmark (2018) to 20–35% in Spain, Portugal, France and Belgium in 2019, and up to 48% in Italy in 2019 (Table 1) [88,89,90,91,92,93,94,95]. These MR rates are associated with a dominance of the MLS_B_ phenotype related to both *erm*(B) and *erm*(TR) and associated with serotypes III, V, Ib and Ia (Table 2) [86,87,88,89,92,93,94,95,96,97]. Recently, an increase of macrolide resistance has been described in Portugal with rates of up to 35% [94] linked with the spread of a recombinant lineage of clonal complex (CC) 1 expressing type Ib serotype. Surveillance studies regarding MR among β-haemolytic streptococci, along with resistance determinants and associated clones, are needed in order to assess the correct empiric treatments for severe invasive diseases and also vaccine development.

**Table 2:** Frequency of macrolide resistance determinants among different geographical regions.

**(a) *Streptococcus pneumoniae***			***erm*(B)**		***erm*(TR)**		***erm*(T)**		***mef*(A/E)**		***erm*(B) + *mef*(A/E)**		
Continent	Period	MR (N)	Mean (%)	Range (%)	Mean (%)	Range (%)	Mean (%)	Range (%)	Mean (%)	Range (%)	Mean (%)	Range (%)	References
Africa	2003–2015	579	46.7	36.0–90.2	-	-	-	-	18.40	6.5–62.5	32.8	0.0–46.4	[98,99,100,101]
America	1995–2012	4070	24.4	9.3–73.7	-	-	-	-	67.90	15.8–81.0	5.9	0.0–52.1	[102,103,104,105,106,107]
Asia	2003–2013	1813	53.6	37.1–76.5	-	-	-	-	26.30	2.0–46.0	12.9	2.0–62.9	[103,108,109,110,111]
Europe	1995–2017	2525	53.0	20.8–93.5	-	-	-	-	67.3	1.7–75.0	6.1	0.0–17.9	[103,112,113,114,115,116,117,118]
Oceania	2003–2005	139	28.8	18.9–32.3	-	-	-	-	36.00	27.4–59.5	34.6	21.6–39.2	[103,119]
**(b) *Streptococcus pyogenes***			***erm*(B)**		***erm*(TR)**		***erm*(T)**		***mef*(A/E)**		***erm*(B) + *mef*(A/E)**		
Continent	Period	MR (N)	Mean (%)	Range (%)	Mean (%)	Range (%)	Mean (%)	Range (%)	Mean (%)	Range (%)	Mean (%)	Range (%)	References
Africa	2000–2013	84	19.0	7.6–57.1	26.1	0.0–38.8	-	-	48.8	14.2–57.6	-	-	[69,120]
America	1996–2017	1128	25.6	4.1–25.7	30.5	0.0–36.9	25.6	0.0–55.0	13.7	0.0–95.6	-	-	[15,55,58,59,60,61,121,122]
Asia	1993–2019	877	54.2	18.6–97.7	3.6	0.0–10.5	-	-	39.7	0.0–80.0	-	-	[62,63,64,66,123,124]
Europe	1995–2016	2873	31.5	5.8–70.9	14.6	0.0–59.0	-	-	40.7	0.0–80.0	-	-	[3,16,17,35,36,39,40,41,43,44,45,47,49,50,51,125,126,127,128]
**(c) *Streptococcus dys s. equisimilis***			***erm*(B)**		***erm*(TR)**		***erm*(T)**		***mef*(A/E)**		***erm*(B) + *mef*(A/E)**		
Continent	Period	MR (N)	Mean (%)	Range (%)	Mean (%)	Range (%)	Mean (%)	Range (%)	Mean (%)	Range (%)	Mean (%)	Range (%)	References
America	1979–2013	30	26.6	12.5–33.3	40.0	25.0–50.0	-	-	33.3	16.7–62.5	-	-	[85,129,130]
Asia	1993–2015	320	31.2	8.5–86.3	9.0	0.0–66.6	-	-	2.8	0.0–64.2	-	-	[62,68,79,80,81,82,131,132]
Europe	2000–2015	154	16.2	5.8–29.1	59.0	35.7–8.7	0.6	0.0–5.8	17.5	0.0–23.5	-	-	[35,49,51,74,75,76]
**(d) *Streptococcus agalactiae***			***erm*(B)**		***erm*(TR)**		***erm*(T)**		***mef*(A/E)**		***erm*(B) + *mef*(A/E)**		
Continent	Period	MR (N)	Mean (%)	Range (%)	Mean (%)	Range (%)	Mean (%)	Range (%)	Mean (%)	Range (%)	Mean (%)	Range (%)	References
Africa	2018–2018	13 *	7.6	-	53.8	-	-	-	23.0	-	-	-	[133]
America	2008–2018	5652	35.0	34.9–64.5	34.0	12.5–70.9	3.20	0.0–16.1	26.9	22.2–54.8	-	-	[88,89,134,135]
Asia	2003–2019	94	70.2	59.3–82.5	22.3	7.5–36.3	-	-	3.1	0.0–5.0	-	-	[68,78,136]
Europe	2000–2019	1219	64.1	35.8–80.9	16.6	0.0–36.6	0.40	0.0–1.0	13.80	2.3–22.0	-	-	[35,46,51,94,95,96,97,137,138]
Oceania	1999–2017	32 *	37.5	-	37.5	-	6.20	-	0.0	-	-	-	[139]

N MR: Number of macrolide-resistant isolates accumulated. * No range of percentages are specified if only one study was found per geographical area per microorganism.

## 4. Macrolide Resistance in *S. pneumoniae*

The prevalence of macrolide resistance in pneumococcus differs depending on numerous factors, such as geographic location (Figure 1, Table 1), selective pressure mediated by antibiotic consumption or the introduction of conjugate vaccines [22]. The first MR *S. pneumoniae* isolates were detected in 1967 in Canada [140], but resistance rates remained low worldwide during the 1970s. The introduction of long-action macrolides resulted in a rapid increase in resistance in many countries [4]. In pneumococci, macrolide resistance is mainly related to the presence of *erm*(B) and/or *mef*(A/E) genes (Table 2) [98,102,108,112,117,118].

Many studies on macrolide resistance have been conducted throughout Europe over the years (Table S1). In the eastern countries, different trends have been observed. In Bulgaria, the rate remained stable between 1995–2005 (18.9%) and 2006–2010 (19.0%) [113], and an increase to 43.9% was observed in 2011–2016 [141]. Contrarily, in Hungary, resistance apparently decreased from 43.6% in 2003–2004 [103] to 25.3% in 2015–2016 [142]. In some areas of northern countries, the resistance rate was very low, as demonstrated in a study in the Faroe Islands, Denmark, between 2009 and 2011 where only 1.5% of isolates from the children’s nasopharynx were resistant [143], or in Skåne, Sweden, where only 8 resistant isolates of 2131 non-invasive pneumococci (0.3%) collected in the 2016–2018 period were found [144]. In the south, the resistance rates remain at around 20% nowadays. In a study conducted in Croatia during 2005 to 2019, 23.0% of pneumococcus causing invasive disease in adults were non-susceptible to macrolides [145]. Lower rates were found in Slovenia, where the resistant rates in invasive disease between 1997 and 2017 were 15.0% [118]. In Spain, a decrease of macrolide resistance was observed between the 2012–2013 and 2015–2016 periods (from 24.0% to 16.3%) among invasive isolates from adults [5]. 

Throughout the 1990s, the spread of multidrug-resistant clones, such as Spain^23F^-1 or Spain^6B^-2 having *erm*(B), or England^14^-9 harboring *mef*(A), were related to macrolide resistance in European countries [4,112]. Throughout the 2000s, with the introduction of the 7-valent pneumococcal conjugate vaccine (PCV7), these multidrug-resistant clones almost disappeared, but macrolide resistance rates remained high, associated with different lineages. For instance, isolates of CC230 expressing serotypes 19A and 24F and harbouring *erm*(B) have been prevalent in Europe, while the CC320^19A^ clone, harboring both *erm*(B) and *mef*(E), was worldwide disseminated [4]. Besides these, other lineages are also contributing to macrolide resistance. In this way, CC63^15A^, CC558^35B^ and CC386^6C^ harboring *erm*(B) or CC100^33F^ harboring *mef*(E) have been found in several European countries [146,147,148,149,150].

Differences between countries are also described in South America. Before PCV7 introduction, resistance rates in Argentina were 12.1% [103], whereas a study carried out by Zintgraff et al. describes a progressive increase from 20.4% in 2006–2008 to 35.2% in 2017–2018 among invasive disease in children [151]. These results are in concordance with a study performed in Bogota, Colombia, where MR rates in children with invasive disease increased from 4.8% in 2008–2011 to 35.2% in 2014–2019 [152]. In Lima, Peru, the resistance rates were higher, at 85.9% in the 2016–2019 period [153]. On the other hand, in Canada (North America), 19.2% of *S. pneumoniae* isolates from invasive and 22.8% from non-invasive sources were resistant (2007–2016) [154].

In the USA, a recent study of ABCs showed a 29.2% rate of resistance among 2881 invasive pneumococci from 2017 [155]. Among them, around 70% had *mef*(E)-*mrs*(D), mainly associated with CC433^22F^, CC538^35B^ and CC100^33F^ lineages. On the other hand, nearly 30% had the *erm*(B) gene alone (CC63^15A^) or associated with *mef*(E)-*mrs*(D) in serotype 19A isolates (CC320). These results were comparable to a previous study performed with 2316 IPD isolates collected in 2015 [156]. Nevertheless, as occurred in other reports, resistance rates were higher among respiratory samples, reaching 47.3% in a national study conducted in adults in 2018–2019, while resistance among isolates from bacteraemia was 29.6% [157].

The highest MR rates are reported from the Asian continent, most notably in the east. In China, 81.6% of pneumococcus isolates between 2003 and 2004 showed macrolide resistance [103]; from 2008 to 2019 rates increased to more than 95% regardless of age [158,159,160,161], type of disease [162] or carriage (Table 1) [109,160,161]. According to a colonization study in children performed in the city of Chongqing in 2020, the MR rate was 56.6%, which is the lowest rate reported in this country [163]. A slightly lower rate was described in a national study conducted in Japan between 2001 and 2015, where the rate was higher in children (84.1%) than adults (75.3%) [164], and in a study from Chungnam, South Korea, where 79.2% of pneumococci showed MR [165]. 

An eleven-year (1998–2014) surveillance study in Casablanca, Morocco, showed low macrolide resistance rates in all periods, despite a slight increase observed from 9.4% in 1998–2001 to 14.0% in 2007–2014 [166]. A recent study conducted in Addis Ababa, Ethiopia, between 2016 and 2017 found that 33.6% of pneumococci were resistant to macrolides [167]. Similar data were described in a national study in Algeria with a resistance rate of 31.0% between 2001 and 2010 [168]. Pneumococcus causing disease in immunocompromised patients in Tunis, Tunisia (2005–2011) presented higher resistance rates with 69.5% of isolates non-susceptible to this antibiotic family [169]. In Australia, 14.8% of *S. pneumoniae* isolated in the children’s nasopharynx (1999–2005) were non-susceptible to macrolides [170], the rate being higher in invasive isolates collected in 2005 (31.0%) [119]. A recent study (2011–2017) showed a reduction in this rate (13.0%) and also in invasive disease in children [171].

## 5. Macrolide Resistance in Other Streptococci

Viridans group streptococci (VGS) belong to the microbiota from the upper respiratory tract, gastrointestinal tract and female genital tract [172]. As commensal microorganisms, they have a low pathogenic potential in immunocompetent individuals, but can cause invasive disease (endocarditis, pneumonia, intra-abdominal infection and shock) [173]. An increasing problem associated with this group is that they can act as a reservoir of resistance genes [174]. It has been demonstrated that bacteria living in biofilms in areas such as the throat readily share genes with each other [175]. For example, some macrolide-resistance genes can be transferred from commensal VGS to pathogenic *S. pyogenes* or *S. pneumoniae* [176,177]. There is concern about the antimicrobial resistance of VGS because of the treatment of its infections, but also because of their role as reservoirs of drug resistance, including mobile genetic elements carrying macrolide resistance determinants [172]. In this way, several studies showed high MR rates among VGS [172,178,179,180]. 

Focusing on macrolides, variable data regarding the resistance mechanisms exist. Some studies found a MLS_B_ phenotype predominance with the *erm*(B) gene [178,181] and others found a M phenotype predominance with the *mef* genes [172,177,180,182].

There are few studies about MR in VGS causing disease that showed an increase in resistance rates over the last three decades as well as changes in the associated phenotypes. In this way, a study of VGS collected from blood cultures in Turkey (1996–2004) found a low resistance rate (27%) with a predominance of the MLS_B_ phenotype due to *erm*(B) [179]. In 2010–2012 a study in Italy about oral VGS in patients with pharyngitis found 56.3% of macrolide resistance, mainly associated with the M phenotype (75%). The most important species carrying this resistance were *Streptococcus mitis*, *Streptococcus oralis*, *Streptococcus parasanguinis*, *Streptococcus sanguinis* and *Streptococcus salivarius*. The most common resistance element found was the macrolide efflux genetic assembly (MEGA) element with *mef*(E) [172]. In line with this, a multicenter study from 12 European countries in 2010–2013 found a 41% macrolide resistance rate among VGS, and 69% of the MR strains showed the M phenotype and 31% the MLS_B_ phenotype [183]. Other non-European studies reported high macrolide resistance rates [148] in VGS collected from sterile sources (Korea 34%, USA 41%, Canada 38% and Northern Taiwan 40%) [178,184,185]. Finally, a study from the USA showed an increase of macrolide resistance between 2010 and 2020 [186]. Other studies explored MR rates and associated genes in VGS carried by healthy people. For instance, a Greek study in healthy children in the late 1990s found 38.5% of macrolide resistance with predominance of the M phenotype (74%), and *S. oralis* showing the highest MR rates [187]. A Finnish study in the elderly found 22.4% of macrolide resistance and 80.6% of the resistant strains had the M phenotype (*mef*(A) gene) [188]. A French study of clinical and commensal *S. salivarius* found 56% and 76% of MR strains, respectively. The predominant phenotype in both samples was the M phenotype [189]. In Belgium, 71% of volunteers between the ages of 17 and 25 years old carried MR VGS with predominance of the MLS_B_ phenotype, while another study in Spain with MR strains from 172 patients showed that the M phenotype with the *mef*(E) gene was predominant in the early 2000s [181,190]. To sum up, MR is highly spread in VGS, as it is shown in different studies around the world. MR rates oscillated between 22% and 76%, but it could be considered a high rate in all samples, age groups and health conditions. The predominant macrolide resistance phenotype was different among the studies, but the M phenotype was explained by the *mef*(A/E) genes and the MLS_B_ phenotype by the *erm*(B) gene in all of them. As these genes could be transferred in MGEs to another bacterial species [14], VGS constitute a resistance reservoir that could endanger the treatment of infections caused by pathogens such as *S. pyogenes* and *S. pneumoniae* [174,176,177].

## 6. Genetic Mobile Elements Related to Macrolide Resistance

Mobile genetic elements, such as integrative and conjugative elements (ICEs), integrative and mobilizable elements (IMEs), plasmids and bacteriophages, are vectors for the transmission of antibiotic resistance in streptococci; they are able to disseminate intra- and interspecies [14,191]. Among them, integrative conjugative elements (ICEs) had all the genes required for conjugation providing the capacity for self-mobilization, while IME do not have all the genes required for conjugation and need the coexistence of an ICE or a conjugative plasmid in the same cell to spread among other streptococci. Most of these elements are characterized by their ability to recombine and to integrate new resistance genes, such as tetracycline, chloramphenicol or aminoglycosides resistance determinants, conferring a multidrug-resistant phenotype. In this way, the role of these MGEs in the dissemination of resistance genes is an important threat to the global emergence of antibiotic resistance in streptococci [14,191,192]. 

Two main *mef* genes (*mef*(A) and *mef(*E)) are found in streptococci. Among them, the *mef*(A) gene is carried into the phage φ1207.3 and is most frequently found in *S. pyogenes.* In this sense, the *mef*(A)-*msr*(D) tandem is found as a necessary signature for the production of an active efflux transport system. Nevertheless, the Tn*1207.1* was associated with the spread of the *mef*(A) gene in pneumococci linked to the international clone England^14^-ST9. On the other hand, the *mef*(E) gene is carried by the MEGA as a *mef*(E)-*mrs*(D) tandem that could also be integrated into larger structures containing other resistance determinants [3,14,192].

Several MGEs have been identified carrying the *erm*(TR) gene, mainly in *S. pyogenes*. Among them, the IME*Sp*2907 and the so-called *erm*(TR)-element are the principal carrier IMEs with different rearrangements and also inserted in larger structures containing other resistance determinants. In this way, the IME*Sp*2907 could be present in the ICE*Sp2905* also carrying *tet*(O), ICE*SpHKU165* structures carrying the *tet*(M) gene as integration of a Tn*916*, or ICE*SagTR7* also carrying *tet*(M) [3,193]. The latter has been found in a mosaic ICE (ICE*Sag236*), which emerged after recombination with the ICE*Spn529IQ*, carrying *mef*(I), and *catQ* and conferring a multidrug-resistant phenotype to *S. agalactiae* [194]. On the other hand, the *erm*(TR)-element has been described inside the ICESp*1108*-like structures that frequently carry *tet*(M) and sporadically *tet*(T). These ICE*Sp1108*-like structures have also been described in *S. agalactiae* and *S. suis*, demonstrating its ability to spread among different streptococci [3,14,193,195].

The *erm*(B) gene is frequently present in macrolide-resistant strains from different streptococcal species, such as *S. pneumoniae*, *S. pyogenes* or *S. agalactiae*. The most important group of elements carrying this gene are transposons of the Tn*916*-family that are characterized by the presence of *tet*(M) [14,192]. Among them, Tn*6002* originated from the insertion of *erm*(B) into the Tn*916* and Tn*6003*, which had a further insertion of the macrolide–aminoglycoside–streptothricin (MAS) element with two *erm*(B) genes; these are the most frequent. On the other hand, the insertion of Tn*917* carrying *erm*(B) into a Tn*916* emerged as the Tn*3872.* These elements are usually located within a Tn*5252*-like structures that frequently carry *cat* gene conferring a multidrug-resistance phenotype. Recently, a new structure (ICE*Sp1070HUB*) carrying *erm*(B) has been described in *S. pyogenes*. This structure also harbors other resistance determinants (*tet*(M), *dfrF*, *cat*(pC194) and the aminoglycoside-modifying enzymes cluster *aph*(3′)III-*sat4*-*ant*(6)Ia) conferring a multidrug-resistant phenotype [3].

The association of *erm*(B) and *mef*(E) genes was related to the spread of dual-macrolide resistance in pneumococci linked to the spread of the CC320^19A^ clone. This dual resistance is carried by the Tn*2010*, which emerged after two recombination events. Firstly, the MEGA element was integrated into Tn*916* becoming the Tn*2009* that further acquired the MAS element carrying the *erm*(B) gene [14]. 

Although the *erm*(T) is sporadically detected in streptococci, its frequency is slightly growing especially in *S. pyogenes*, SDSE and *S. agalactiae*. In these species, the resistance gene was located on small non-self-transmissible plasmids that could be transferred into *trans* profiting from ICE*Sde3396*-like structures [196]. 

## 7. Macrolide Consumption

The emergence and spread of antibiotic resistance is closely associated with antibiotic use. Antibiotics act as a selection pressure that favors the spread of resistant bacteria, either by promoting the spread of pre-existing resistant bacteria or by selecting resistance acquired during the course of treatment. In fact, macrolide consumption has been directly associated with the prevalence of macrolide-resistant *S. pneumoniae* [197], and this may have important clinical consequences [198]. It also seems that macrolide resistance associated with previous exposure could last longer than that caused by other antimicrobial groups [199]. In the same line, experiences with mass drug administration of azithromycin for trachoma control show that this has a direct impact on the increase of macrolide-resistant *S. pneumoniae* carriage [200,201]. Therefore, in addition to the study of the clonal composition of bacteria and the molecular basis of antimicrobial resistance, the study of antimicrobial consumption is also key to understanding the dynamics of antimicrobial resistance.

The consumption of antimicrobials for human use increased worldwide during the 2000–2010 period, mainly due to a strong increase in countries with rapid economic expansion, such as India or China [202]. Despite regional differences, macrolide antibiotics were the third most frequent class sold in 2010, up slightly from 2000. In Europe, macrolide consumption for human use in the community has remained stable over the last years [203]. For example, macrolide consumption changed from 3.2 to 2.9 DDD (defined daily dose) per 1000 inhabitants per day in Spain over the period of 1997–2017. This stability in macrolide consumption has been accompanied by a substantial change in the type of macrolide prescribed. The consumption of short-acting macrolides (i.e., erythromycin) has decreased dramatically (from 0.89 to 0.13 DDD per 1000 inhabitants per day), while the consumption of long-acting macrolides (i.e., azithromycin) has increased (0.55 to 2.03 DDD per 1000 inhabitants per day). In the USA, data on macrolide consumption showed a decreasing trend over the past 10 years, but also with a clear predominance of azithromycin as the most prescribed drug (190 and 90 macrolide prescriptions per 1000 persons and 170 and 86 azithromycin prescriptions per 1000 persons in 2011 and 2021, respectively) [204,205]. In China, the consumption of macrolides in hospitals increased slightly over the period of 2011–2015 (from 1.19 to 1.41 DDDs per 1000 inhabitants per day) [206] with a predominance of roxithromycin (intermediate-acting), clarithromycin and azithromycin as the most used macrolides in 2017 [207]. It should be noted that long-term exposure to azithromycin or erythromycin has been reported to increase the proportion of macrolide resistance in oropharyngeal streptococci [208]. In this study, the proportion of macrolide-resistant streptococci remained higher in patients that received azithromycin when compared to patients that received erythromycin after antibiotic cessation. Then, it is plausible that the pattern of antibiotic replacement from short- to long-acting macrolides has had a direct impact on the selection of macrolide resistance. 

Besides human use, antimicrobials are extensively used in food-producing animals, which also supposes a risk for selecting antimicrobial resistance in bacteria [209]. Moreover, food-producing animals can be a source of human infections caused by resistant bacteria [210]. Macrolide antibiotics have been categorized among the highest-priority critically important antimicrobials by the WHO, requiring measures to reduce the risk of transmission of resistance to humans [211]. The EMA-AMEG (European Medicines Agency, Antimicrobial Advice ad hoc Expert Group) classified macrolides in category C (“caution”) as there are some indications in veterinary medicine with few or no alternatives to macrolides [212]. Global consumption of antimicrobials in food-producing animals has been estimated at 63,151 tonnes in 2010 and is expected to increase to 105,596 tonnes in 2030 due to increased numbers of raised animals [213]. In 2017, China was the largest antimicrobial consumer in food-producing animals, accounting for 45% of global consumption [214]. Despite these estimations, declines in antimicrobial sales have been reported in high-income countries in recent years. Data on antimicrobial consumption in 29 European countries in 2017 showed that the total amount of antimicrobials consumed was superior in food-producing animals than in humans (6558 vs. 4122 tonnes). When adjusted per Kg of estimated biomass, macrolides were the third and the second most frequent group of antimicrobials used among food-producing animals (median 5.7 mg/kg estimated biomass) and humans (median 6.4 mg/kg estimated biomass), respectively [215]. In 2020, macrolide consumption accounted for 8.8% of all veterinary antimicrobial prescriptions in EU/EEA countries and, more importantly, a clear downward trend (mg/PCU) was evident for most antimicrobials, including macrolides, over the last nine years (2011–2020) [216]. In the USA, macrolides were the third most frequent group of medically important antimicrobials marketed in 2020 (7%, total amount of 433,394 kg) and showed a decreasing trend since 2011 (−26% compared to 2020), similar to what occurred with other antimicrobial groups [217]. All these data evidence the effectiveness of the measures adopted to reduce the consumption of antimicrobials in food-producing animals in some countries [218]. It should be noted that interventions to restrict antibiotic use in food-producing animals have been associated with a decrease of antibiotic-resistant bacteria in animals [219]. In an ecological study, a positive association between the use of macrolides in food-producing animals and macrolide resistance in *S. pneumoniae* isolates of human origin has been reported; although this should be interpreted with caution, taking into account all the limitations of this type of study [218]. It has also been described that exposure of soil bacteria to a high macrolide concentration can alter the resistome composition, leading to an increased number of resistance genes to multiple antibiotic classes [48]. Therefore, measures to limit the consumption of medically important antimicrobials, such as macrolides, in food-producing animals are essential to prevent the emergence and dissemination of resistant bacteria.

## 8. Conclusions

In conclusion, macrolide resistance in streptococci varies between countries and species. Over the last decades, surveillance of resistance rates and the genetic studies have helped us to understand the dynamics of resistance among species. In this way, several aspects contribute to the spread of resistance including the dissemination of mobile genetic elements, the antibiotic pressure exerted by consumption in humans and animals, the impact of the introduction of vaccines and the spread of resistant lineages. The dissemination of MGEs carrying resistance determinants among different streptococcal species is a cause for concern and deserves further surveillance. Furthermore, an in-depth analysis of reservoirs of resistance, including non-pathogenic streptococci as well as those causing disease in animals, could be an important step to improve the understanding of resistance dissemination.

## Supplementary Materials

The following supporting information can be downloaded at: https://www.mdpi.com/article/10.3390/microorganisms10122316/s1, Table S1 (a–d): Review of the literature of macrolide resistance rates among different geographical regions: (a) *Streptococcus pneumoniae*; (b) *Streptococcus pyogenes*; (c) *Streptococcus dysgalactiae* subsp *equisimilis*; (d) *Streptococcus agalactiae*.

## References

[1] Patel S., Gupta R.S. (2018). Robust Demarcation of Fourteen Different Species Groups within the Genus *Streptococcus* Based on Genome-Based Phylogenies and Molecular Signatures. Infect. Genet. Evol..

[2] Lannes-Costa P.S., de Oliveira J.S.S., da Silva Santos G., Nagao P.E. (2021). A Current Review of Pathogenicity Determinants of *Streptococcus* Sp.. J. Appl. Microbiol..

[3] Berbel D., Càmara J., González-Díaz A., Cubero M., López De Egea G., Martí S., Tubau F., Domínguez M.A., Ardanuy C. (2021). Deciphering Mobile Genetic Elements Disseminating Macrolide Resistance in *Streptococcus pyogenes* over a 21 Year Period in Barcelona, Spain. J. Antimicrob. Chemother..

[4] Liñares J., Ardanuy C., Pallares R., Fenoll A. (2010). Changes in Antimicrobial Resistance, Serotypes and Genotypes in *Streptococcus pneumoniae* over a 30-Year Period. Clin. Microbiol. Infect..

[5] González-Díaz A., Càmara J., Ercibengoa M., Cercenado E., Larrosa N., Quesada M.D., Fontanals D., Cubero M., Marimón J.M., Yuste J. (2020). Emerging Non-13-Valent Pneumococcal Conjugate Vaccine (PCV13) Serotypes Causing Adult Invasive Pneumococcal Disease in the Late-PCV13 Period in Spain. Clin. Microbiol. Infect..

[6] Wilson D.N. (2014). Ribosome-Targeting Antibiotics and Mechanisms of Bacterial Resistance. Nat. Rev. Microbiol..

[7] Roberts M.C., Sutcliffe J., Courvalin P., Bogo Jensen L., Rood J., Seppala H. (1999). Nomenclature for Macrolide and Macrolide-Lincosamide-Streptogramin B Resistance Determinants. Antimicrob. Agents Chemother..

[8] Crowe-McAuliffe C., Murina V., Turnbull K.J., Kasari M., Mohamad M., Polte C., Takada H., Vaitkevicius K., Johansson J., Ignatova Z. (2021). Structural Basis of ABCF-Mediated Resistance to Pleuromutilin, Lincosamide, and Streptogramin A Antibiotics in Gram-Positive Pathogens. Nat. Commun..

[9] World Health Organization (2016). Critically Important Antimicrobials for Human Medicine—5th Rev.

[10] Ferretti J.J., Stevens D.L., Fischetti V.A. (2016). Streptococcus pyogenes Basic Biology to Clinical Manifestations.

[11] Versalovic J., Carroll K.C., Funke G., Jorgensen J.H., Landry M.L., Warnock D.W. (2015). Manual of Clinical Microbiology.

[12] Sharkey L.K.R., O’Neill A.J. (2018). Antibiotic Resistance ABC-F Proteins: Bringing Target Protection into the Limelight. ACS Infect. Dis..

[13] Sutcliffe J., Tait-Kamradt A., Wondrack L. (1996). *Streptococcus pneumoniae* and *Streptococcus pyogenes* Resistant to Macrolides but Sensitive to Clindamycin: A Common Resistance Pattern Mediated by an Efflux System. Antimicrob. Agents Chemother..

[14] Varaldo P.E., Montanari M.P., Giovanetti E. (2009). Genetic Elements Responsible for Erythromycin Resistance in Streptococci. Antimicrob. Agents Chemother..

[15] DiPersio L.P., DiPersio J.R. (2007). Identification of an *Erm*(T) Gene in Strains of Inducibly Clindamycin-Resistant Group B *Streptococcus*. Diagn. Microbiol. Infect. Dis..

[16] Silva-Costa C., Friães A., Ramirez M., Melo-Cristino J. (2015). Macrolide-Resistant *Streptococcus pyogenes*: Prevalence and Treatment Strategies. Expert. Rev. Antimicrob. Infect..

[17] Ardanuy C., Domenech A., Rolo D., Calatayud L., Tubau F., Ayats J., Martín R., Liñares J. (2010). Molecular Characterization of Macrolide-and Multidrug-Resistant *Streptococcus pyogenes* Isolated from Adult Patients in Barcelona, Spain (1993–2008). J. Antimicrob. Chemother..

[18] Kataja J., SeppälA H., Skurnik M., Sarkkinen H., Huovinen P. (1998). Different Erythromycin Resistance Mechanisms in Group C and Group G Streptococci. Antimicrob. Agents Chemother..

[19] Jalava J., Vaara M., Huovinen P. (2004). Mutation at the Position 2058 of the 23S RRNA as a Cause of Macrolide Resistance in *Streptococcus pyogenes*. Ann. Clin. Microbiol. Antimicrob..

[20] Malbruny B., Nagai K., Coquemont M., Bozdogan B., Andrasevic A.T., Hupkova H., Leclercq R., Appelbaum P.C. (2002). Resistance to Macrolides in Clinical Isolates of *Streptococcus pyogenes* Due to Ribosomal Mutations. J. Antimicrob. Chemother..

[21] Farrell D.J., Shackcloth J., Barbadora K.A., Green M.D. (2006). *Streptococcus pyogenes* Isolates with High-Level Macrolide Resistance and Reduced Susceptibility to Telithromycin Associated with 23S RRNA Mutations. Antimicrob. Agents Chemother..

[22] Schroeder M.R., Stephens D.S. (2016). Macrolide Resistance in *Streptococcus pneumoniae*. Front. Cell Infect. Microbiol..

[23] Reinert R.R., Wild A., Appelbaum P.C., Lütticken R., Cil M.Y., Al-Lahham A. (2003). Ribosomal Mutations Conferring Resistance to Macrolides in *Streptococcus pneumoniae* Clinical Strains Isolated in Germany. Antimicrob. Agents Chemother..

[24] Bozdogan B., Appelbaum P.C., Ednie L., Grivea I.N., Syrogiannopoulos G.A. (2002). Development of Macrolide Resistance by Ribosomal Protein L4 Mutation in *Streptococcus pyogenes* during Miocamycin Treatment of an Eight-Year-Old Greek Child with Tonsillopharyngitis. Clin. Microbiol. Infect..

[25] Sangvik M., Littauer P., Simonsen G.S., Sundsfjord A., Dahl K.H. (2005). *Mef*(A), *Mef*(E) and a New *Mef* Allele in Macrolide-Resistant *Streptococcus* Spp. Isolates from Norway. J. Antimicrob. Chemother..

[26] Cochetti I., Vecchi M., Mingoia M., Tili E., Catania M.R., Manzin A., Varaldo P.E., Montanari M.P. (2005). Molecular Characterization of Pneumococci with Efflux-Mediated Erythromycin Resistance and Identification of a Novel Mef Gene Subclass, *Mef*(I). Antimicrob. Agents Chemother..

[27] Iannelli F., Santoro F., Santagati M., Docquier J.D., Lazzeri E., Pastore G., Cassone M., Oggioni M.R., Rossolini G.M., Stefani S. (2018). Type M Resistance to Macrolides Is Due to a Two-Gene Efflux Transport System of the ATP-Binding Cassette (ABC) Superfamily. Front. Microbiol..

[28] Gay K., Stephens D.S. (2000). Structure and Dissemination of a Chromosomal Insertion Element Encoding Macrolide Efflux in *Streptococcus pneumoniae*. J. Infect. Dis..

[29] Ardanuy C., Tubau F., Liñares J., Domínguez M.A., Pallarés R., Martín R. (2005). Distribution of Subclasses *MefA* and *MefE* of the *MefA* Gene among Clinical Isolates of Macrolide-Resistant (M-Phenotype) *Streptococcus pneumoniae*, Viridans Group Streptococci, and *Streptococcus pyogenes*. Antimicrob. Agents Chemother..

[30] Brenciani A., Bacciaglia A., Vignaroli C., Pugnaloni A., Varaldo P.E., Giovanetti E. (2010). Φm46.1, the Main *Streptococcus pyogenes* Element Carrying *Mef*(A) and *Tet*(O) Genes. Antimicrob. Agents Chemother..

[31] Santagati M., Iannelli F., Oggioni M.R., Stefani S., Pozzi G. (2000). Characterization of a Genetic Element Carrying the Macrolide Efflux Gene *Mef*(A) in *Streptococcus pneumoniae*. Antimicrob. Agents Chemother..

[32] Sharkey L.K.R., Edwards T.A., O’Neill A.J. (2016). ABC-F Proteins Mediate Antibiotic Resistance through Ribosomal Protection. mBio.

[33] Eugene S., Foster M.T., Scott D. (1969). Group A Beta-Hemolytic Streptococci Resistant to Erythromycin and Lincomycin. N. Engl. J. Med..

[34] Montes M., Ardanuy C., Tamayo E., Domènech A., Liñares J., Pérez-Trallero E. (2011). Epidemiological and Molecular Analysis of *Streptococcus pyogenes* Isolates Causing Invasive Disease in Spain (1998-2009): Comparison with Non-Invasive Isolates. Eur. J. Clin. Microbiol. Infect. Dis..

[35] Merino-Díaz L., Torres-Sánchez M.J., Aznar-Martín J. (2008). Prevalence and Mechanisms of Erythromycin and Clindamycin Resistance in Clinical Isolates of β-Haemolytic Streptococci of Lancefield Groups A, B, C and G in Seville, Spain. Clin. Microbiol. Infect..

[36] Montes M., Tamayo E., Mojica C., García-Arenzana J.M., Esnal O., Pérez-Trallero E. (2014). What Causes Decreased Erythromycin Resistance in *Streptococcus pyogenes*? Dynamics of Four Clones in a Southern European Region from 2005 to 2012. J. Antimicrob. Chemother..

[37] Calle-Miguel L., Perez-Mendez C., Miguel-Martinez M.D., Lombraña-Alvarez E., Garcia-Garcia E., Solis-Sanchez G. (2005). Resistance Rates and Phenotypic Characterization of *Streptococcus pyogenes* in a Paediatric Population in Northern Spain. Rev. Española Quimioter..

[38] Villalón P., Sáez-Nieto J.A., Rubio-López V., María J.M.P., Medina-Pascual J., Garrido N., Carrasco G., Pino-Rosa S., Valdezate S. (2021). Invasive *Streptococcus pyogenes* Disease in Spain: A Microbiological and Epidemiological Study Covering the Period 2007-2019. Eur. J. Clin. Microbiol. Infect. Dis..

[39] Friães A., Pinto F.R., Silva-Costa C., Ramirez M., Melo-Cristino J. (2012). Group A Streptococci Clones Associated with Invasive Infections and Pharyngitis in Portugal Present Differences in *Emm* Types, Superantigen Gene Content and Antimicrobial Resistance. BMC Microbiol..

[40] Friães A., Melo-Cristino J., Ramirez M., Vaz T., Gião M., Ferreira R., Fonseca A.B., Oliveira H., Silva A.C., Costa H. (2019). Changes in *Emm* Types and Superantigen Gene Content of *Streptococcus pyogenes* Causing Invasive Infections in Portugal. Sci. Rep..

[41] Michos A.G., Bakoula C.G., Braoudaki M., Koutouzi F.I., Roma E.S., Pangalis A., Nikolopoulou G., Kirikou E., Syriopoulou V.P. (2009). Macrolide Resistance in *Streptococcus pyogenes*: Prevalence, Resistance Determinants, and *Emm* Types. Diagn. Microbiol. Infect. Dis..

[42] Malli E., Tatsidou E., Damani A., Pantelidi K., Petinaki E. (2010). Macrolide-Resistant *Streptococcus pyogenes* in Central Greece: Prevalence; Mechanism and Molecular Identification. Int. J. Antimicrob. Agents.

[43] Grivea I.N., Syrogiannopoulos G.A., Michoula A.N., Gazeti G., Malli E., Tsilipounidaki K., Fouzas S., Anthracopoulos M.B., Petinaki E. (2020). *Emm* Types and Clusters and Macrolide Resistance of Pediatric Group A Streptococcal Isolates in Central Greece during 2011–2017. PLoS ONE.

[44] Olivieri R., Morandi M., Zanchi A., Tordini G., Pozzi G., de Luca A., Montagnani F. (2015). Evolution of Macrolide Resistance in *Streptococcus pyogenes* over 14 Years in an Area of Central Italy. J. Med. Microbiol..

[45] D’Humières C., Cohen R., Levy C., Bidet P., Thollot F., Wollner A., Bingen E. (2012). Decline in Macrolide-Resistant *Streptococcus pyogenes* Isolates from French Children. Int. J. Med. Microbiol..

[46] Smit P.W., Lindholm L., Lyytikäinen O., Jalava J., Pätäri-Sampo A., Vuopio J. (2015). Epidemiology and *Emm* Types of Invasive Group A Streptococcal Infections in Finland, 2008–2013. Eur. J. Clin. Microbiol. Infect. Dis..

[47] Meehan M., Murchan S., Gavin P.J., Drew R.J., Cunney R. (2018). Epidemiology of an Upsurge of Invasive Group A Streptococcal Infections in Ireland, 2012–2015. J. Infect..

[48] Brown L.P., Murray R., Scott A., Tien Y.C., Lau C.H.F., Tai V., Topp E. (2022). Responses of the Soil Bacterial Community, Resistome, and Mobilome to a Decade of Annual Exposure to Macrolide Antibiotics. Appl. Environ. Microbiol..

[49] Amezaga M.R., McKenzie H. (2006). Molecular Epidemiology of Macrolide Resistance in β-Haemolytic Streptococci of Lancefield Groups A, B, C and G and Evidence for a New Mef Element in Group G Streptococci That Carries Allelic Variants of *Mef* and *Msr*(D). J. Antimicrob. Chemother..

[50] Imöhl M., van der Linden M. (2015). Antimicrobial Susceptibility of Invasive *Streptococcus pyogenes* Isolates in Germany during 2003–2013. PLoS ONE.

[51] Van Leer Buter C.C., Mouton J.W., Klaassen C.H.W., Handgraaf C.M.A., Sunnen S., Melchers W.J.G., Sturm P.D.J. (2010). Prevalence and Molecular Mechanism of Macrolide Resistance in β-Haemolytic Streptococci in The Netherlands. Int. J. Antimicrob. Agents.

[52] Biedenbach D.J., Toleman M.A., Walsh T.R., Jones R.N. (2006). Characterization of Fluoroquinolone-Resistant β-Hemolytic *Streptococcus* Spp. Isolated in North America and Europe Including the First Report of Fluoroquinolone-Resistant *Streptococcus dysgalactiae* Subspecies Equisimilis: Report from the SENTRY Antimicrobial Surveillance Program (1997–2004). Diagn. Microbiol. Infect. Dis..

[53] De Muri G.P., Sterkel A.K., Kubica P.A., Duster M.N., Reed K.D., Wald E.R. (2017). Macrolide and Clindamycin Resistance in Group a Streptococci Isolated from Children with Pharyngitis. Pediatr. Infect. Dis. J..

[54] Fay K., Onukwube J., Chochua S., Schaffner W., Cieslak P., Lynfield R., Muse A., Smelser C., Harrison L.H., Farley M. (2021). Patterns of Antibiotic Nonsusceptibility among Invasive Ggroup A *Streptococcus* Infections*—*United States, 2006–2017. Clin. Infect. Dis..

[55] Chochua S., Metcalf B.J., Li Z., Rivers J., Mathis S., Jackson D., Gertz R.E., Srinivasan V., Lynfield R., van Beneden C. (2017). Population and Whole Genome Sequence Based Characterization of Invasive Group a Streptococci Recovered in the United States during 2015. mBio.

[56] Sanson M.A., Macias O.R., Shah B.J., Hanson B., Vega L.A., Alamarat Z., Flores A.R. (2019). Unexpected Relationships between Frequency of Antimicrobial Resistance, Disease Phenotype and Emm Type in Group a *Streptococcus*. Microb. Genom..

[57] Li Y., Rivers J., Mathis S., Li Z., McGee L., Chochua S., Metcalf B.J., Fleming-Dutra K.E., Nanduri S.A., Beall B. (2022). Continued Increase of Erythromycin- and Clindamycin-Nonsusceptibility among Invasive Group A *Streptococci* Driven by Genomic Clusters, USA, 2018–2019. Clin. Infect. Dis..

[58] Rodríguez C., Rojas P., Wozniak A., Kalergis A.M., Cerón I., Riedel I., Román J.C., Villarroel L.A., Berríos X., Bavestrello L. (2011). Resistance Phenotypes and Genotypes of *Streptococcus pyogenes* Clinical Isolatesin Chile over a 10-Year Period. Rev. Med. Chil..

[59] Villaseñor-Sierra A., Katahira E., Jaramillo-Valdivia A.N., de los Angeles Barajas-García M., Bryant A., Morfín-Otero R., Márquez-Díaz F., Tinoco J.C., Sánchez-Corona J., Stevens D.L. (2012). Phenotypes and Genotypes of Erythromycin-Resistant *Streptococcus pyogenes* Strains Isolated from Invasive and Non-Invasive Infections from Mexico and the USA during 1999-2010. Int. J. Infect. Dis..

[60] Rubinstein G., Amoroso A.M., Bavdaz B., de Bunder S., Blazquez N., Gutkind G.O. (2005). Low Macrolide Resistance in *Streptococcus pyogenes* in Southern Argentina. Int. J. Antimicrob. Agents.

[61] Arêas G.P., Schuab R.B.B., Neves F.P.G., Barros R.R. (2014). Antimicrobial Susceptibility Patterns, Emm Type Distribution and Genetic Diversity of *Streptococcus pyogenes* Recovered in Brazil. Mem. Inst. Oswaldo Cruz.

[62] Ip M., Lyon D., Leung T., Cheng A. (2002). Macrolide Resistance and Distribution of *Erm* and *Mef*Genes among Beta-Haemolytic Streptococci in Hong Kong. Eur. J. Clin. Microbiol. Infect. Dis..

[63] Lu B., Fang Y., Fan Y., Chen X., Wang J., Zeng J., Li Y., Zhang Z., Huang L., Li H. (2017). High Prevalence of Macrolide-Resistance and Molecular Characterization of *Streptococcus pyogenes* Isolates Circulating in China from 2009 to 2016. Front. Microbiol..

[64] Tsai W.C., Shen C.F., Lin Y.L., Shen F.C., Tsai P.J., Wang S.Y., Lin Y.S., Wu J.J., Chi C.Y., Liu C.C. (2021). Emergence of Macrolide-Resistant *Streptococcus pyogenes Emm*12 in Southern Taiwan from 2000 to 2019. J. Microbiol. Immunol. Infect..

[65] Tatara K., Gotoh K., Okumiya K., Teramachi M., Ishimoto K., Tanaka Y., Iwahashi J., Shindou S., Yamashita Y., Watanabe H. (2020). Molecular Epidemiology, Antimicrobial Susceptibility, and Characterization of Fluoroquinolone Non-Susceptible *Streptococcus pyogenes* in Japan. J. Infect. Chemother..

[66] Ikebe T., Okuno R., Kanda Y., Sasaki M., Yamaguchi T., Otsuka H., Kazawa Y., Suzuki M., Ohya H., Uchida K. (2021). Molecular Characterization and Antimicrobial Resistance of Group A *Streptococcus* Isolates in Streptococcal Toxic Shock Syndrome Cases in Japan from 2013 to 2018. Int. J. Med. Microbiol..

[67] Cho Y.N., Park S.E., Cho E.Y., Cho H.K., Park J.Y., Kang H.-M., Yun K.W., Choi E.H., Lee H. (2022). Distribution of *Emm* Genotypes in Group A *Streptococcus* Isolates of Korean Children from 2012 to 2019. J. Microbiol. Immunol. Infect..

[68] Uh Y., Hwang G.Y., Jang I.H., Cho H.M., Noh S.M., Kim N.Y., Kwon O., Yoon K.J. (2007). Macrolide Resistance Trends in β-Hemolytic Streptococci in a Tertiary Korean Hospital. Yonsei Med. J..

[69] Rafei R., Hawli M., Osman M., Dabboussi F., Hamze M. (2020). Distribution of *Emm* Types and Macrolide Resistance Determinants among Group A Streptococci in the Middle East and North Africa Region. J. Glob. Antimicrob. Resist..

[70] Canetti M., Carmi A., Paret G., Goldberg L., Adler A., Amit S., Rokney A., Ron M., Grisaru-Soen G. (2021). Invasive Group A *Streptococcus* Infection in Children in Central Israel in 2012-2019. Pediatr. Infect. Dis. J..

[71] Barsenga S., Mitiku H., Tesfa T., Shume T. (2022). Throat Carriage Rate, Associated Factors, and Antimicrobial Susceptibility Pattern of Group A *Streptococcus* among Healthy School Children in Jigjiga City, Eastern Ethiopia. BMC Pediatr..

[72] Foster-Nyarko E., Kwambana B., Ceesay F., Jawneh K., Darboe S., Mulwa S.N., Ceesay B., Secka O.O., Adetifa I., Antonio M. (2017). Incidence of Macrolide-Lincosamide-Streptogramin B Resistance amongst Beta-Haemolytic Streptococci in the Gambia. BMC Res. Notes.

[73] Haenni M., Lupo A., Madec J.-Y. (2018). Antimicrobial Resistance in *Streptococcus* spp.. Microbiol Spectr.

[74] Rojo-Bezares B., Toca L., Manuel Azcona-Gutiérrez J., Ortega-Unanue N., Toledano P., Sáenz Y. (2021). *Streptococcus dysgalactiae* Subsp. Equisimilis from Invasive and Non-Invasive Infections in Spain: Combining Epidemiology, Molecular Characterization, and Genetic Diversity. Eur. J. Clin. Microbiol. Infect. Dis..

[75] Loubinoux J., Plainvert C., Collobert G., Touak G., Bouvet A., Poyart C. (2013). Adult Invasive and Noninvasive Infections Due to *Streptococcus dysgalactiae* Subsp. Equisimilis in France from 2006 to 2010. J. Clin. Microbiol..

[76] Gherardi G., Imperi M., Palmieri C., Magi G., Facinelli B., Baldassarri L., Pataracchia M., Creti R. (2013). Genetic Diversity and Virulence Properties of *Streptococcus dysgalactiae* Subsp. Equisimilis from Different Sources. J. Med. Microbiol..

[77] Leitner E., Zollner-Schwetz I., Zarfel G., Masoud-Landgraf L., Gehrer M., Wagner-Eibel U., Grisold A.J., Feierl G. (2015). Prevalence of *Emm* Types and Antimicrobial Susceptibility of *Streptococcus dysgalactiae* Subsp. Equisimilis in Austria. Int. J. Med. Microbiol..

[78] Park J.H., Jung J., Kim M.J., Sung H., Kim M.N., Chong Y.P., Kim S.H., Lee S.O., Kim Y.S., Woo J.H. (2019). Incidence, Clinical Characteristics, and Outcomes of *Streptococcus dysgalactiae* Subspecies Equisimilis Bacteremia in a Tertiary Hospital: Comparison with *S. Agalactiae* Bacteremia. Eur. J. Clin. Microbiol. Infect. Dis..

[79] Lu B., Fang Y., Huang L., Diao B., Du X., Kan B., Cui Y., Zhu F., Li D., Wang D. (2016). Molecular Characterization and Antibiotic Resistance of Clinical *Streptococcus dysgalactiae* Subsp. Equisimilis in Beijing, China. Infect. Genet. Evol..

[80] Lo H.H., Nien H.H., Cheng Y.Y., Su F.Y. (2015). Antibiotic Susceptibility Pattern and Erythromycin Resistance Mechanisms in Beta-Hemolytic Group G *Streptococcus dysgalactiae* Subspecies Equisimilis Isolates from Central Taiwan. J. Microbiol. Immunol. Infect..

[81] Zheng P.X., Chan Y.C., Chiou C.S., Hsieh C.L., Chiang-Ni C., Wu J.J. (2017). Highly Prevalent *EmmSTG840.0* and *EmmSTC839.0* Types of Erythromycin Non-Susceptible Group G *Streptococcus* Isolated from Bacteremia in Southern Taiwan. J. Microbiol. Immunol. Infect..

[82] Wajima T., Morozumi M., Hanada S., Sunaoshi K., Chiba N., Iwata S., Ubukata K. (2016). Molecular Characterization of Invasive *Streptococcus dysgalactiae* Subsp. Equisimilis, Japan. Emerg. Infect. Dis..

[83] Arnold B., Bélard S., Alabi A., Hufnagel M., Berner R., Toepfner N. (2022). High Diversity of *Emm* Types and Marked Tetracycline Resistance of Group A Streptococci and Other ß-Hemolytic Streptococci in Gabon, Central Africa. Pediatr. Infect. Dis. J..

[84] Halperin T., Levine H., Korenman Z., Burstein S., Amber R., Sela T., Valinsky L. (2016). Molecular Characterization and Antibiotic Resistance of Group G Streptococci in Israel: Comparison of Invasive, Non-Invasive and Carriage Isolates. Eur. J. Clin. Microbiol. Infect. Dis..

[85] De Souza J.P., Santos A.R., de Paula G.R., Barros R.R. (2016). Antimicrobial Susceptibility and Genetic Relationships among *Streptococcus dysgalactiae* Subsp. Equisimilis Isolates in Rio de Janeiro. Infect. Dis..

[86] Guo D., Cao X., Li S., Ou Q., Lin D., Yao Z., Chen S., Wu C., Wen G., Ye X. (2018). Neonatal Colonization of Group B *Streptococcus* in China: Prevalence, Antimicrobial Resistance, Serotypes, and Molecular Characterization. Am. J. Infect. Control.

[87] Bae H.G., Hong J., Kim Y.-J., Lee K.-R., Lee K., Choi S.J., Uh Y. (2022). A Retrospective National Study on Colonization Rate and Antimicrobial Susceptibility of *Streptococcus agalactiae* in Pregnant Korean Women, 2018–2020. Yonsei Med. J..

[88] McGee L., Chochua S., Li Z., Mathis S., Rivers J., Metcalf B., Ryan A., Alden N., Farley M.M., Harrison L.H. (2021). Multistate, Population-Based Distributions of Candidate Vaccine Targets, Clonal Complexes, and Resistance Features of Invasive Group B Streptococci within the United States, 2015–2017. Clin. Infect. Dis..

[89] Metcalf B.J., Chochua S., Gertz R.E., Hawkins P.A., Ricaldi J., Li Z., Walker H., Tran T., Rivers J., Mathis S. (2017). Short-Read Whole Genome Sequencing for Determination of Antimicrobial Resistance Mechanisms and Capsular Serotypes of Current Invasive *Streptococcus agalactiae* Recovered in the USA. Clin. Microbiol. Infect..

[90] Slotved H.C., Hoffmann S. (2020). The Epidemiology of Invasive Group B *Streptococcus* in Denmark from 2005 to 2018. Front. Public Health.

[91] Graux E., Hites M., Martiny D., Maillart E., Delforge M., Melin P., Dauby N. (2021). Invasive Group B *Streptococcus* among Non-Pregnant Adults in Brussels-Capital Region, 2005–2019. Eur. J. Clin. Microbiol. Infect. Dis..

[92] Genovese C., D’Angeli F., di Salvatore V., Tempera G., Nicolosi D. (2020). *Streptococcus agalactiae* in Pregnant Women: Serotype and Antimicrobial Susceptibility Patterns over Five Years in Eastern Sicily (Italy). Eur. J. Clin. Microbiol. Infect. Dis..

[93] Vuillemin X., Hays C., Plainvert C., Dmytruk N., Louis M., Touak G., Saint-Pierre B., Adoux L., Letourneur F., Frigo A. (2021). Invasive Group B *Streptococcus Infections* in Non-Pregnant Adults: A Retrospective Study, France, 2007–2019. Clin. Microbiol. Infect..

[94] Lopes E., Fernandes T., Machado M.P., Carriço J.A., Melo-Cristino J., Ramirez M., Martins E.R., Oliveira H., Vaz T., Gião M. (2018). Increasing Macrolide Resistance among *Streptococcus agalactiae* Causing Invasive Disease in Non-Pregnant Adults Was Driven by a Single Capsular-Transformed Lineage, Portugal, 2009 to 2015. Eurosurveillance.

[95] López Y., Parra E., Cepas V., Sanfeliú I., Juncosa T., Andreu A., Xercavins M., Pérez J., Sanz S., Vergara A. (2018). Serotype, Virulence Profile, Antimicrobial Resistance and Macrolide-Resistance Determinants in *Streptococcus agalactiae* Isolates in Pregnant Women and Neonates in Catalonia, Spain. Enferm. Infect. Microbiol. Clin..

[96] Kekic D., Gajic I., Opavski N., Kojic M., Vukotic G., Smitran A., Boskovic L., Stojkovic M., Ranin L. (2021). Trends in Molecular Characteristics and Antimicrobial Resistance of Group B Streptococci: A Multicenter Study in Serbia, 2015–2020. Sci. Rep..

[97] Plainvert C., Hays C., Touak G., Joubrel-Guyot C., Dmytruk N., Frigo A., Poyart C., Tazi A. (2020). Multidrug-Resistant Hypervirulent Group B *Streptococcus* in Neonatal Invasive Infections, France, 2007–2019. Emerg. Infect. Dis..

[98] Diawara I., Zerouali K., Katfy K., Barguigua A., Belabbes H., Timinouni M., Elmdaghri N. (2016). Phenotypic and Genotypic Characterization of *Streptococcus pneumoniae* Resistant to Macrolide in Casablanca, Morocco. Infect. Genet. Evol..

[99] Taha N., Araj G.F., Wakim R.H., Kanj S.S., Kanafani Z.A., Sabra A., Khairallah M.T., Nassar F.J., Shehab M., Baroud M. (2012). Genotypes and Serotype Distribution of Macrolide Resistant Invasive and Non- Invasive *Streptococcus pneumoniae* Isolates from Lebanon. Ann. Clin. Microbiol. Antimicrob..

[100] El Ashkar S., Osman M., Rafei R., Mallat H., Achkar M., Dabboussi F., Hamze M. (2017). Molecular Detection of Genes Responsible for Macrolide Resistance among *Streptococcus pneumoniae* Isolated in North Lebanon. J. Infect. Public Health.

[101] Azadegan A., Ahmadi A., Lari A.R., Talebi M. (2015). Detection of the Efflux-Mediated Erythromycin Resistance Transposon in *Streptococcus pneumoniae*. Ann. Lab. Med..

[102] Rudolph K., Bulkow L., Bruce M., Zulz T., Reasonover A., Harker-Jones M., Hurlburt D., Hennessy T. (2013). Molecular Resistance Mechanisms of Macrolide-Resistant Invasive *Streptococcus pneumoniae* Isolates from Alaska, 1986 to 2010. Antimicrob. Agents Chemother..

[103] Farrell D.J., Couturier C., Hryniewicz W. (2008). Distribution and Antibacterial Susceptibility of Macrolide Resistance Genotypes in *Streptococcus pneumoniae*: PROTEKT Year 5 (2003–2004). Int. J. Antimicrob. Agents.

[104] Wierzbowski A.K., Nichol K., Laing N., Hisanaga T., Nikulin A., Karlowsky J.A., Hoban D.J., Zhanel G.G. (2007). Macrolide Resistance Mechanisms among *Streptococcus pneumoniae* Isolated over 6 Years of Canadian Respiratory Organism Susceptibility Study (CROSS) (1998–2004). J. Antimicrob. Chemother..

[105] Bowers J.R., Driebe E.M., Nibecker J.L., Wojack B.R., Sarovich D.S., Wong A.H., Brzoska P.M., Hubert N., Knadler A., Watson L.M. (2012). Dominance of Multidrug Resistant CC271 Clones in Macrolide-Resistant *Streptococcus pneumoniae* in Arizona. BMC Microbiol.

[106] Reijtman V., Gagetti P., Faccone D., Fossati S., Eck P.S., Hernández C., Bernáldez P., Lopardo H., Corso A. (2013). Macrolide Resistance in Streptococcus Pneumoniae Isolated from Argentinian Pediatric Patients Suffering from Acute Otitis Media. Rev. Argent. Microbiol..

[107] Caierão J., Hawkins P., Sant’anna F.H., da Cunha G.R., D’Azevedo P.A., McGee L., Dias C. (2014). Serotypes and Genotypes of Invasive *Streptococcus pneumoniae* before and after PCV10 Implementation in Southern Brazil. PLoS ONE.

[108] Okade H., Funatsu T., Eto M., Furuya Y., Mizunaga S., Nomura N., Mitsuyama J., Yamagishi Y., Mikamo H. (2014). Impact of the Pneumococcal Conjugate Vaccine on Serotype Distribution and Susceptibility Trends of Pediatric Non-Invasive Streptococcus Pneumoniae Isolates in Tokai, Japan over a 5-Year Period. J. Infect. Chemother..

[109] Geng Q., Zhang T., Ding Y., Tao Y., Lin Y., Wang Y., Black S., Zhao G. (2014). Molecular Characterization and Antimicrobial Susceptibility of *Streptococcus pneumoniae* Isolated from Children Hospitalized with Respiratory Infections in Suzhou, China. PLoS ONE.

[110] Mayanskiy N., Alyabieva N., Ponomarenko O., Lazareva A., Katosova L., Ivanenko A., Kulichenko T., Namazova-Baranova L., Baranov A. (2014). Serotypes and Antibiotic Resistance of Non-Invasive *Streptococcus pneumoniae* Circulating in Pediatric Hospitals in Moscow, Russia. Int. J. Infect. Dis..

[111] Sirekbasan L., Gönüllü N., Sirekbasan S., Kuşkucu M., Midilli K. (2015). Phenotypes and Genotypes of Macrolide-Resistant *Streptococcus pneumoniae*. Balk. Med. J..

[112] Calatayud L., Ardanuy C., Tubau F., Rolo D., Grau I., Pallarés R., Martín R., Linares J. (2010). Serotype and Genotype Replacement among Macrolide-Resistant Invasive Pneumococci in Adults: Mechanisms of Resistance and Association with Different Transposons. J. Clin. Microbiol..

[113] Setchanova L.P., Alexandrova A., Mitov I., Nashev D., Kantardjiev T. (2012). Serotype Distribution and Antimicrobial Resistance of Invasive *Streptococcus pneumoniae* Isolates in Bulgaria before the Introduction of Pneumococcal Conjugate Vaccine. J. Chemother..

[114] De la Pedrosa E.G.G., Baquero F., Loza E., Nadal-Serrano J.M., Fenoll A., del Campo R., Cantón R. (2009). High Clonal Diversity in Erythromycin-Resistant Streptococcus Pneumoniae Invasive Isolates in Madrid, Spain (2000–07). J. Antimicrob. Chemother..

[115] Siira L., Rantala M., Jalava J., Hakanen A.J., Huovinen P., Kaijalainen T., Lyytikäinen O., Virolainen A. (2009). Temporal Trends of Antimicrobial Resistance and Clonality of Invasive *Streptococcus pneumoniae* Isolates in Finland, 2002 to 2006. Antimicrob. Agents Chemother..

[116] Bley C., van der Linden M., Reinert R.R. (2011). *Mef*(A) Is the Predominant Macrolide Resistance Determinant in *Streptococcus pneumoniae* and *Streptococcus pyogenes* in Germany. Int. J. Antimicrob. Agents.

[117] Lismond A., Carbonnelle S., Verhaegen J., Schatt P., de Bel A., Jordens P., Jacobs F., Dediste A., Verschuren F., Huang T.D. (2012). Antimicrobial Susceptibility of *Streptococcus pneumoniae* Isolates from Vaccinated and Non-Vaccinated Patients with a Clinically Confirmed Diagnosis of Community-Acquired Pneumonia in Belgium. Int. J. Antimicrob. Agents.

[118] Kastrin T., Paragi M., Erčulj V., Žohar Čretnik T., Bajec T., Čižman M. (2019). Lack of Correlation between Reduced Outpatient Consumption of Macrolides and Macrolide Resistance of Invasive *Streptococcus pneumoniae* Isolates in Slovenia during 1997–2017. J. Glob. Antimicrob. Resist..

[119] Xu X., Cai L., Xiao M., Kong F., Oftadeh S., Zhou F., Gilbert G.L. (2010). Distribution of Serotypes, Genotypes, and Resistance Determinants among Macrolide-Resistant *Streptococcus pneumoniae* Isolates. Antimicrob. Agents Chemother..

[120] Ksia S., Smaoui H., Hraoui M., Bouafsoun A., Boutiba-Ben Boubaker I., Kechrid A. (2017). Molecular Characteristics of Erythromycin-Resistant *Streptococcus pyogenes* Strains Isolated from Children Patients in Tunis, Tunisia. Microb. Drug Resist..

[121] Li Y., Rivers J., Mathis S., Li Z., Velusamy S., Nanduri S.A., van Beneden C.A., Snippes-Vagnone P., Lynfield R., McGee L. (2020). Genomic Surveillance of *Streptococcus pyogenes* Strains Causing Invasive Disease, United States, 2016–2017. Front. Microbiol..

[122] Mendes C., Marin M.E., Quiñones F., Súcari A., Rossi F., Barriga Angulo G., Segura A., Starling C., Mimica Brasília I., Francisco de Assis Hospital S. (2003). Antibacterial Resistance of Community-Acquired Respiratory Tract Pathogens Recovered from Patients in Latin America: Results from the PROTEKT Surveillance Study (1999–2000). Braz. J. Infect. Dis..

[123] Abraham T., Sistla S. (2018). Trends in Antimicrobial Resistance Patterns of Group A Streptococci, Molecular Basis and Implications. Indian J. Med. Microbiol..

[124] Tanaka Y., Gotoh K., Teramachi M., Ishimoto K., Tsumura N., Shindou S., Yamashita Y. (2016). Molecular Epidemiology, Antimicrobial Susceptibility, and Characterization of Macrolide-Resistant *Streptococcus pyogenes* in Japan. J. Infect. Chemother..

[125] Pérez-Trallero E., Tamayo E., Montes M., García-Arenzana J.M., Iriarte V. (2011). In Vitro Activities of Retapamulin and 16 Other Antimicrobial Agents against Recently Obtained *Streptococcus pyogenes* Isolates. Antimicrob. Agents Chemother..

[126] Farmand S., Henneke P., Hufnagel M., Berner R. (2012). Significant Decline in the Erythromycin Resistance of Group A *Streptococcus* Isolates at a German Paediatric Tertiary Care Centre. Eur. J. Clin. Microbiol. Infect. Dis..

[127] Muhtarova A.A., Gergova R.T., Mitov I.G. (2017). Distribution of Macrolide Resistance Mechanisms in Bulgarian Clinical Isolates of *Streptococcus pyogenes* during the Years of 2013–2016. J. Glob. Antimicrob. Resist..

[128] Littauer P., Caugant D.A., Sangvik M., Høiby E.A., Sundsfjord A., Simonsen G.S. (2006). Macrolide-Resistant *Streptococcus pyogenes* in Norway: Population Structure and Resistance Determinants. Antimicrob. Agents Chemother..

[129] Silva L.G., Genteluci G.L., de Mattos M.C., Glatthardt T., Sà Figueiredo A.M., Ferreira-Carvalho B.T. (2015). Group C *Streptococcus dysgalactiae* Subsp. Equisimilis in South-East Brazil: Genetic Diversity, Resistance Profile and the First Report of Human and Equine Isolates Belonging to the Same Multilocus Sequence Typing Lineage. J. Med. Microbiol..

[130] Traverso F., Blanco A., Villalón P., Beratz N., Sáez Nieto J.A., Lopardo H. (2016). Molecular Characterization of Invasive *Streptococcus dysgalactiae* Subsp. Equisimilis. Multicenter Study: Argentina 2011–2012. Rev. Argent. Microbiol..

[131] Fujita T., Horiuchi A., Ogawa M., Yoshida H., Hirose Y., Nagano N., Takahashi T. (2017). Genetic Diversity in *Streptococcus dysgalactiae* Subsp. Equisimilis Isolates from Patients with Invasive and Noninvasive Infections in a Japanese University Hospital (2014–2015). Jpn. J. Infect. Dis..

[132] Sunaoshi K., Murayama S.Y., Adachi K., Yagoshi M., Okuzumi K., Chiba N., Morozumi M., Ubukata K. (2010). Molecular *Emm* Genotyping and Antibiotic Susceptibility of *Streptococcus dysgalactiae* Subsp. Equisimilis Isolated from Invasive and Non-Invasive Infections. J. Med. Microbiol..

[133] Bob-Manuel M., McGee L., Igunma J.A., Alex-Wele M.A., Obunge O.K., Wariso K.T. (2021). Whole Genome Sequence Based Capsular Typing and Antimicrobial Resistance Prediction of Group B Streptococcal Isolates from Colonized Pregnant Women in Nigeria. BMC Genom..

[134] Santana F.A.F., de Oliveira T.V.L., de Souza Filho M.B., da Silva L.S.C., de Brito B.B., de Melo F.F., Souza C.L., Marques L.M., Oliveira M.V. (2020). Streptococcus agalactiae: Identification Methods, Antimicrobial Susceptibility, and Resistance Genes in Pregnant Women. World J. Clin. Cases.

[135] DiPersio L.P., DiPersio J.R., Beach J.A., Loudon A.M., Fuchs A.M. (2011). Identification and Characterization of Plasmid-Borne *Erm*(T) Macrolide Resistance in Group B and Group A *Streptococcus*. Diagn. Microbiol. Infect. Dis..

[136] Kawaguchiya M., Urushibara N., Aung M.S., Shimada S., Nakamura M., Ito M., Habadera S., Kobayashi N. (2022). Molecular Characterization and Antimicrobial Resistance of *Streptococcus agalactiae* Isolated from Pregnant Women in Japan, 2017–2021. IJID Reg..

[137] Artiles Campelo F., Cañas Pedrosa A., Álamo Antúnez I., Lafarga Capuz B. (2012). Phenotypes and Mechanisms of Resistance to Macrolides and Lincosamides in *Streptococcus agalactiae* Isolates with Clinical Significance in an Eight-Year Period. Rev. Española Quimioter..

[138] Dobrut A., Ochońska D., Brzozowska E., Górska S., Kaszuba-Zwoinska J., Gołda-Cępa M., Gamian A., Brzychczy-Wloch M. (2022). Molecular Characteristic, Antibiotic Resistance, and Detection of Highly Immunoreactive Proteins of Group B *Streptococcus* Strains Isolated from Urinary Tract Infections in Polish Adults. Front. Microbiol..

[139] Jones S., Newton P., Payne M., Furfaro L. (2022). Epidemiology, Antimicrobial Resistance, and Virulence Determinants of Group B *Streptococcus* in an Australian Setting. Front. Microbiol..

[140] Dixon J.M.S. (1967). Pneumococcus resistant to erythromycin and lincomycin. Lancet.

[141] Setchanova L., Murdjeva M., Stancheva I., Alexandrova A., Sredkova M., Stoeva T., Yoneva M., Kurchatova A., Mitov I. (2017). Serotype Changes and Antimicrobial Nonsusceptibility Rates of Invasive and Non-Invasive *Streptococcus pneumoniae* Isolates after Implementation of 10-Valent Pneumococcal Nontypeable Haemophilus Influenzae Protein D Conjugate Vaccine (PHiD-CV) in Bulgaria. Braz. J. Infect. Dis..

[142] Kovács E., Sahin-Tóth J., Tóthpál A., Kristóf K., van der Linden M., Tirczka T., Dobay O. (2019). Vaccine-Driven Serotype-Rearrangement Is Seen with Latency in Clinical Isolates: Comparison of Carried and Clinical Pneumococcal Isolates from the Same Time Period in Hungary. Vaccine.

[143] Debess Magnussen M., Erlendsdóttir H., Gaini S., Gudnason T., Kristinsson K.G. (2018). *Streptococcus pneumoniae*: Antimicrobial Resistance and Serotypes of Strains Carried by Children and Causing Invasive Disease in the Faroe Islands. Microb. Drug Resist..

[144] Uddén F., Rünow E., Slotved H.C., Fuursted K., Ahl J., Riesbeck K. (2021). Characterization of *Streptococcus pneumoniae* Detected in Clinical Respiratory Tract Samples in Southern Sweden 2 to 4 Years after Introduction of PCV13. J. Infect..

[145] Butić I., Gužvinec M., Jelić M., Groš I., Lucić S., Bošnjak M., Tambić Andrašević A. (2022). Serotype Distribution and Antimicrobial Resistance of Invasive *Streptococcus pneumoniae* Isolates among Croatian Adults during a Fifteen-Year Period (2005–2019). Croat. Med. J..

[146] González-Díaz A., Berbel D., Ercibengoa M., Cercenado E., Larrosa N., Quesada M.D., Casabella A., Cubero M., Marimón J.M., Domínguez M.Á. (2022). Genomic Features of Predominant Non-PCV13 Serotypes Responsible for Adult Invasive Pneumococcal Disease in Spain. J. Antimicrob. Chemother..

[147] Lo S.W., Mellor K., Cohen R., Alonso A.R., Belman S., Kumar N., Hawkins P.A., Gladstone R.A., von Gottberg A., Veeraraghavan B. (2022). Emergence of a Multidrug-Resistant and Virulent *Streptococcus pneumoniae* Lineage Mediates Serotype Replacement after PCV13: An International Whole-Genome Sequencing Study. Lancet Microbe.

[148] Janoir C., Cohen R., Levy C., Bingen E., Lepoutre A., Gutmann L., Varon E., Ploy M.C., Baraduc R., Brun M. (2014). Clonal Expansion of the Macrolide Resistant ST386 within Pneumococcal Serotype 6C in France. PLoS ONE.

[149] Càmara J., Grau I., González-Diáz A., Tubau F., Calatayud L., Cubero M., Domínguez M.Á., Linãres J., Yuste J., Pallarés R. (2021). A Historical Perspective of MDR Invasive Pneumococcal Disease in Spanish Adults. J. Antimicrob. Chemother..

[150] Horácio A.N., Silva-Costa C., Diamantino-Miranda J., Lopes J.P., Ramirez M., Melo-Cristino J., Vaz T., Gião M., Ferreira R., Fonseca A.B. (2016). Population Structure of *Streptococcus pneumoniae* Causing Invasive Disease in Adults in Portugal before PCV13 Availability for Adults: 2008–2011. PLoS ONE.

[151] Zintgraff J., Gagetti P., Napoli D., Sanchez Eluchans N., Irazu L., Moscoloni M., Regueira M., Lara C.S., Corso A. (2022). Invasive *Streptococcus pneumoniae* Isolates from Pediatric Population in Argentina for the Period 2006–2019. Temporal Progression of Serotypes Distribution and Antibiotic Resistance. Vaccine.

[152] Gutiérrez-Tobar I.F., Londoño-Ruiz J.P., Mariño-Drews C., Beltrán-Higuera S., Camacho-Moreno G., Leal-Castro A.L., Patiño-Niño J.A., Álvarez-Olmos M.I., Barrero-Barreto R., Espinosa F. (2022). Epidemiological Characteristics and Serotype Distribution of Culture-Confirmed Pediatric Pneumococcal Pneumonia before and after PCV10 Introduction, a Multicenter Study in Bogota, Colombia, 2008–2019. Vaccine.

[153] Gonzales B.E., Mercado E.H., Pinedo-Bardales M., Hinostroza N., Campos F., Chaparro E., del Águila O., Castillo M.E., Saenz A., Reyes I. (2022). Increase of Macrolide-Resistance in *Streptococcus pneumoniae* Strains after the Introduction of the 13-Valent Pneumococcal Conjugate Vaccine in Lima, Peru. Front. Cell Infect. Microbiol..

[154] Golden A.R., Baxter M.R., Davidson R.J., Martin I., Demczuk W., Mulvey M.R., Karlowsky J.A., Hoban D.J., Zhanel G.G., Adam H.J. (2019). Comparison of Antimicrobial Resistance Patterns in *Streptococcus pneumoniae* from Respiratory and Blood Cultures in Canadian Hospitals from 2007–16. J. Antimicrob. Chemother..

[155] Varghese J., Chochua S., Tran T., Walker H., Li Z., Snippes Vagnone P.M., Lynfield R., McGee L., Li Y., Metcalf B.J. (2020). Multistate Population and Whole Genome Sequence-Based Strain Surveillance of Invasive Pneumococci Recovered in the USA during 2017. Clin. Microbiol. Infect..

[156] Metcalf B.J., Chochua S., Gertz R.E., Li Z., Walker H., Tran T., Hawkins P.A., Glennen A., Lynfield R., Li Y. (2016). Using Whole Genome Sequencing to Identify Resistance Determinants and Predict Antimicrobial Resistance Phenotypes for Year 2015 Invasive Pneumococcal Disease Isolates Recovered in the United States. Clin. Microbiol. Infect..

[157] Gupta V., Yu K.C., Schranz J., Gelone S.P. (2021). A Multicenter Evaluation of the US Prevalence and Regional Variation in Macrolide-Resistant *S. pneumoniae* in Ambulatory and Hospitalized Adult Patients in the United States. Open Forum Infect Dis.

[158] Kim S.H., Song J.H., Chung D.R., Thamlikitkul V., Yang Y., Wang H., Lu M., So T.M.K., Hsueh P.R., Yasin R.M. (2012). Changing Trends in Antimicrobial Resistance and Serotypes of *Streptococcus pneumoniae* Isolates in Asian Countries: An Asian Network for Surveillance of Resistant Pathogens (ANSORP) Study. Antimicrob. Agents Chemother..

[159] Zhao C., Li Z., Zhang F., Zhang X., Ji P., Zeng J., Hu B., Hu Z., Liao K., Sun H. (2017). Serotype Distribution and Antibiotic Resistance of *Streptococcus pneumoniae* Isolates from 17 Chinese Cities from 2011 to 2016. BMC Infect. Dis..

[160] Zhou X., Liu J., Zhang Z., Cui B., Wang Y., Zhang Y., Xu H., Cheng G., Liu Y., Qin X. (2022). Characterization of *Streptococcus pneumoniae* Macrolide Resistance and Its Mechanism in Northeast China over a 20-Year Period. Microbiol. Spectr..

[161] Peng S., Ren H., Deng J., Zhao N., Li Y., Li M., Yuan Q., Zhang Z., Luo L., Zeng L. (2021). Genotypic and Phenotypic Characteristics of *Streptococcus pneumoniae* from Community-Acquired Pneumonia Patients and Healthy Asymptomatic Participants in Sichuan Province, China. BMC Infect Dis.

[162] Wang C.Y., Chen Y.H., Fang C., Zhou M.M., Xu H.M., Jing C.M., Deng H.L., Cai H.J., Jia K., Han S.Z. (2019). Antibiotic Resistance Profiles and Multidrug Resistance Patterns of *Streptococcus pneumoniae* in Pediatrics: A Multicenter Retrospective Study in Mainland China. Medicine.

[163] Lei L., Wang X. (2022). Determining the Frequency of *Streptococcus pneumoniae* Carriers and Its Microbial Resistance in Children. Cell Mol. Biol..

[164] Toda H., Satoh K., Komatsu M., Fukuda S., Nakamura T., Jikimoto T., Nishio H., Yamasaki K., Maede T., Orita T. (2018). Laboratory Surveillance of Antimicrobial Resistance and Multidrug Resistance among *Streptococcus pneumoniae* Isolated in the Kinki Region of Japan, 2001–2015. J. Infect. Chemother..

[165] Kim J.S., Jung B.K., Kim J.W., Kim G.Y. (2021). Prevalence and Antimicrobial Susceptibility of *Streptococcus pneumoniae* Isolated from Clinical Samples in the Past 8 Years in Korea. Biomed Res Int.

[166] Benbachir M., Elmdaghri N., Belabbes H., Haddioui G., Benzaid H., Zaki B. (2012). Eleven-Year Surveillance of Antibiotic Resistance in *Streptococcus pneumoniae* in Casablanca (Morocco). Microb. Drug Resist..

[167] Negash A.A., Asrat D., Abebe W., Aseffa A., Vaneechoutte M. (2021). Phenotypic and Molecular Characterization of Penicillin and Macrolide-Resistant *Streptococcus pneumoniae* Serotypes among Pediatric Patients in Addis Ababa, Ethiopia. Infect. Drug Resist..

[168] Tali-Maamar H., Laliam R., Bentchouala C., Touati D., Sababou K., Azrou S., Azzam M., Amhis W., Oussadou L., Belouni R. (2012). Serotyping and Antibiotic Susceptibility of *Streptococcus pneumoniae* Strains Isolated in Algeria from 2001 to 2010. Med. Mal. Infect..

[169] Raddaoui A., Simoes A.S., Baaboura R., Felix S., Achour W., Othman T.B., Bejaoui M., Sa-Leao R., Hassen A. (2015). ben Serotype Distribution, Antibiotic Resistance and Clonality of *Streptococcus pneumoniae* Isolated from Immunocompromised Patients in Tunisia. PLoS ONE.

[170] Dunne E.M., Carville K., Riley T.V., Bowman J., Leach A.J., Cripps A.W., Murphy D., Jacoby P., Lehmann D. (2016). Aboriginal and Non-Aboriginal Children in Western Australia Carry Different Serotypes of Pneumococci with Different Antimicrobial Susceptibility Profiles. Pneumonia.

[171] Hernstadt H., Cheung A., Hurem D., Vasilunas N., Phuong L.K., Quinn P., Agrawal R., Daley A.J., Cole T., Gwee A. (2020). Changing Epidemiology and Predisposing Factors for Invasive Pneumococcal Disease at Two Australian Tertiary Hospitals. Pediatr. Infect. Dis. J..

[172] Brenciani A., Tiberi E., Tili E., Mingoia M., Palmieri C., Varaldo P.E., Giovanetti E. (2014). Genetic Determinants and Elements Associated with Antibiotic Resistance in Viridans Group Streptococci. J. Antimicrob. Chemother..

[173] Doern C.D., Burnham C.A.D. (2010). It’s Not Easy Being Green: The Viridans Group Streptococci, with a Focus on Pediatric Clinical Manifestations. J. Clin. Microbiol..

[174] Bryskier A. (2002). Viridans Group Streptococci: A Reservoir of Resistant Bacteria in Oral Cavities. Clin. Microbiol. Infect..

[175] Li Y.H., Lau P.C.Y., Lee J.H., Ellen R.P., Cvitkovitch D.G. (2001). Natural Genetic Transformation of *Streptococcus mutans* Growing in Biofilms. J. Bacteriol..

[176] Jönsson M., Swedberg G. (2006). Macrolide Resistance Can Be Transferred by Conjugation from Viridans Streptococci to *Streptococcus pyogenes*. Int. J. Antimicrob. Agents.

[177] Cerdá-Zolezzi P., Millán-Laplana L., Rubio-Calvo C., Goñi-Cepero P., Canales-Erazo M., Gómez-Lus R. (2004). Molecular Basis of Resistance to Macrolides and Other Antibiotics in Commensal Viridans Group Streptococci and *Gemella* Spp. and Transfer of Resistance Genes to *Streptococcus pneumoniae*. Antimicrob. Agents Chemother..

[178] Uh Y., Shin D.H., Hwang G.Y., Lee M.K., Yoon K.J., Kim H.Y. (2004). Antimicrobial Susceptibility Patterns and Macrolide Resistance Genes of Viridans Group Streptococci from Blood Cultures in Korea. J. Antimicrob. Chemother..

[179] Ergin A., Ercis S., Hasçelik G. (2006). Macrolide Resistance Mechanisms and in Vitro Susceptibility Patterns of Viridans Group Streptococci Isolated from Blood Cultures. J. Antimicrob. Chemother..

[180] Ioannidou S., Papaparaskevas J., Tassios P.T., Foustoukou M., Legakis N.J., Vatopoulos A.C. (2003). Prevalence and Characterization of the Mechanisms of Macrolide, Lincosamide and Streptogramin Resistance in Viridans Group Streptococci. Int. J. Antimicrob. Agents.

[181] Malhotra-Kumar S., Lammens C., Martel A., Mallentjer C., Chapelle S., Verhoeven J., Wijdooghe M., Haesebrouck F., Goossens H. (2004). Oropharyngeal Carriage of Macrolide-Resistant Viridans Group Streptococci: A Prevalence Study among Healthy Adults in Belgium. J. Antimicrob. Chemother..

[182] Tazumi A., Maeda Y., Goldsmith C.E., Coulter W.A., Mason C., Millar B.C., Mccalmont M., Rendall J., Elborn J.S., Matsuda M. (2009). Molecular Characterization of Macrolide Resistance Determinants [*Erm*(B) and *Mef*(A)] in *Streptococcus pneumoniae* and Viridans Group Streptococci (VGS) Isolated from Adult Patients with Cystic Fibrosis (CF). J. Antimicrob. Chemother..

[183] Mendes R.E., Castanheira M., Farrell D.J., Flamm R.K., Sader H.S., Jones R.N. (2017). Prevalence of Macrolide–Lincosamide Resistance and Multidrug Resistance Phenotypes in Streptococcal Isolates Causing Infections in European Hospitals: Evaluation of the in Vitro Activity of Oritavancin and Comparator Agents. J. Glob. Antimicrob. Resist.

[184] Diekema D.J., Beach M.L., Pfaller M.A., Jones R.N., Sentry T., Group P. (2001). Antimicrobial Resistance in Viridans Group Streptococci among Patients with and without the Diagnosis of Cancer in the USA, Canada and Latin America. Clin. Microbiol. Infect..

[185] Teng L.-J., Hsuehb P.-R., Chenb Y.-C., Hoaand S.-W., Luh K.-T. (1998). Antimicrobial Susceptibility of Viridans Group Streptococci in Taiwan with an Emphasis on the High Rates of Resistance to Penicillin and Macrolides in *Streptococcus oralis*. J. Antimicrob. Chemother..

[186] Singh N., Poggensee L., Huang Y., Evans C.T., Suda K.J., Bulman Z.P. (2022). Antibiotic Susceptibility Patterns of Viridans Group Streptococci Isolates in the United States from 2010 to 2020. JAC Antimicrob. Resist..

[187] Ioannidou S., Tassios P.T., Kotsovili-Tseleni A., Foustoukou M., Legakis N.J., Vatopoulos A. (2001). Antibiotic Resistance Rates and Macrolide Resistance Phenotypes of Viridans Group Streptococci from the Oropharynx of Healthy Greek Children. Int. J. Antimicrob. Agents.

[188] Seppälä H., Haanperä M., Al-Juhaish M., Järvinen H., Jalava J., Huovinen P. (2003). Antimicrobial Susceptibility Patterns and Macrolide Resistance Genes of Viridans Group Streptococci from Normal Flora. J. Antimicrob. Chemother..

[189] Chaffanel F., Charron-Bourgoin F., Libante V., Leblond-Bourget N., Payot S. (2015). Resistance Genes and Genetic Elements Associated with Antibiotic Resistance in Clinical and Commensal Isolates of *Streptococcus salivarius*. Appl. Environ. Microbiol..

[190] Zolezzi P.C., Calvo M.C.R., Millán L., Goñi P., Canales M., Capilla S., Durán E., Gómez-Lus R. (2004). Macrolide Resistance Phenotypes of Commensal Viridans Group Streptococci and *Gemella* Spp. and PCR Detection of Resistance Genes. Int. J. Antimicrob. Agents.

[191] Bellanger X., Payot S., Leblond-Bourget N., Guédon G. (2014). Conjugative and Mobilizable Genomic Islands in Bacteria: Evolution and Diversity. FEMS Microbiol. Rev..

[192] Ambroset C., Coluzzi C., Guédon G., Devignes M.D., Loux V., Lacroix T., Payot S., Leblond-Bourget N. (2016). New Insights into the Classification and Integration Specificity of *Streptococcus* Integrative Conjugative Elements through Extensive Genome Exploration. Front. Microbiol..

[193] Mingoia M., Morici E., Marini E., Brenciani A., Giovanetti E., Varaldo P.E. (2016). Macrolide Resistance Gene *Erm*(TR) and *Erm*(TR)-Carrying Genetic Elements in *Streptococcus agalactiae*: Characterization of ICE*Sag*TR7, a New Composite Element Containing IME*Sp*2907. J. Antimicrob. Chemother..

[194] Morici E., Simoni S., Brenciani A., Giovanetti E., Varaldo P.E., Mingoia M. (2017). A New Mosaic Integrative and Conjugative Element from *Streptococcus agalactiae* Carrying Resistance Genes for Chloramphenicol (*CatQ*) and Macrolides [*Mef*(I) and *Erm*(TR)]. J. Antimicrob. Chemother..

[195] Huang J., Ma J., Shang K., Hu X., Liang Y., Li D., Wu Z., Dai L., Chen L., Wang L. (2016). Evolution and Diversity of the Antimicrobial Resistance Associated Mobilome in *Streptococcus Suis*: A Probable Mobile Genetic Elements Reservoir for Other Streptococci. Front Cell Infect. Microbiol..

[196] Palmieri C., Magi G., Creti R., Baldassarri L., Imperi M., Gherardi G., Facinelli B. (2013). Interspecies Mobilization of an *Erm*(T)-Carrying Plasmid of *Streptococcus dysgalactiae* Subsp. Equisimilis by a Coresident ICE of the ICES*A2603* Family. J. Antimicrob. Chemother..

[197] Albrich W.C., Monnet D.L., Harbarth S. (2004). Antibiotic Selection Pressure and Resistance in *Streptococcus pneumoniae* and *Streptococcus pyogenes*. Emerg. Infect. Dis..

[198] Lonks J.R., Garau J., Gomez L., Xercavins M., Ochoa De Echagü A., Gareen I.F., Reiss P.T., Medeiros A.A. (2002). Failure of Macrolide Antibiotic Treatment in Patients with Bacteremia Due to Erythromycin-Resistant *Streptococcus pneumoniae*. Clin. Infect. Dis..

[199] Kuster S.P., Rudnick W., Shigayeva A., Green K., Baqi M., Gold W.L., Lovinsky R., Muller M.P., Powis J.E., Rau N. (2014). Previous Antibiotic Exposure and Antimicrobial Resistance in Invasive Pneumococcal Disease: Results from Prospective Surveillance. Clin. Infect. Dis..

[200] Coles C.L., Mabula K., Seidman J.C., Levens J., Mkocha H., Munoz B., Mfinanga S.G., West S. (2013). Mass Distribution of Azithromycin for Trachoma Control Is Associated with Increased Risk of Azithromycin-Resistant *Streptococcus pneumoniae* Carriage in Young Children 6 Months after Treatment. Clin. Infect. Dis..

[201] Hart J.D., Samikwa L., Meleke H., Burr S.E., Cornick J., Kalua K., Bailey R.L. (2022). Prevalence of Nasopharyngeal *Streptococcus pneumoniae* Carriage and Resistance to Macrolides in the Setting of Azithromycin Mass Drug Administration: Analysis from a Cluster-Randomised Controlled Trial in Malawi, 2015–2017. Lancet Microbe.

[202] Van Boeckel T.P., Gandra S., Ashok A., Caudron Q., Grenfell B.T., Levin S.A., Laxminarayan R. (2014). Global Antibiotic Consumption 2000 to 2010: An Analysis of National Pharmaceutical Sales Data. Lancet Infect. Dis..

[203] Adriaenssens N., Bruyndonckx R., Versporten A., Hens N., Monnet D.L., Molenberghs G., Goossens H., Weist K., Coenen S. (2021). Consumption of Macrolides, Lincosamides and Streptogramins in the Community, European Union/European Economic Area, 1997–2017. J. Antimicrob. Chemother..

[204] (2011). Centers for Disease Control and Prevention Outpatient Antibiotic Prescriptions—United States. https://www.cdc.gov/antibiotic-use/data/report-2011.html.

[205] (2020). Centers for Disease Control and Prevention Outpatient Antibiotic Prescriptions—United States. https://www.cdc.gov/antibiotic-use/data/report-2021.html.

[206] Wushouer H., Tian Y., Guan X.D., Han S., Shi L.W. (2017). Trends and Patterns of Antibiotic Consumption in China’s Tertiary Hospitals: Based on a 5 Year Surveillance with Sales Records, 2011–2015. PLoS ONE.

[207] Liu W., Gillani A.H., Xu S., Chen C., Chang J., Yang C., Ji W., Jiang M., Zhao M., Fang Y. (2021). Antibiotics (Macrolides and Lincosamides) Consumption Trends and Patterns in China′s Healthcare Institutes. Based on a 3 Year Procurement Records, 2015–2017. Int. J. Env. Res. Public Health.

[208] Burr L.D., Taylor S.L., Richard A., Schreiber V., Lingman S., Martin M., Papanicolas L.E., Choo J.M., Rogers G.B. (2022). Assessment of Long-Term Macrolide Exposure on the Oropharyngeal Microbiome and Macrolide Resistance in Healthy Adults and Consequences for Onward Transmission of Resistance. Antimicrob. Agents Chemother..

[209] Chantziaras I., Boyen F., Callens B., Dewulf J. (2014). Correlation between Veterinary Antimicrobial Use and Antimicrobial Resistance in Food-Producing Animals: A Report on Seven Countries. J. Antimicrob. Chemother..

[210] Lazarus B., Paterson D.L., Mollinger J.L., Rogers B.A. (2015). Do Human Extraintestinal *Escherichia coli* Infections Resistant to Expanded-Spectrum Cephalosporins Originate from Food-Producing Animals? A Systematic Review. Clin. Infect. Dis..

[211] Collignon P.C., Conly J.M., Andremont A., McEwen S.A., Aidara-Kane A., Griffin P.M., Agerso Y., Dang Ninh T., Donado-Godoy P., Fedorka-Cray P. (2016). World Health Organization Ranking of Antimicrobials According to Their Importance in Human Medicine: A Critical Step for Developing Risk Management Dtrategies to Vontrol Antimicrobial Tesistance from Food Animal Production. Clin. Infect. Dis..

[212] European Medicines Agency Categorisation of Antibiotics in the European Union. https://www.ema.europa.eu/en/documents/report/categorisation-antibiotics-european-union-answer-request-european-commission-updating-scientific_en.pdf.

[213] Van Boeckel T.P., Brower C., Gilbert M., Grenfell B.T., Levin S.A., Robinson T.P., Teillant A., Laxminarayan R. (2015). Global Trends in Antimicrobial Use in Food Animals. Proc. Natl. Acad. Sci. USA.

[214] Tiseo K., Huber L., Gilbert M., Robinson T.P., van Boeckel T.P. (2020). Global Trends in Antimicrobial Use in Food Animals from 2017 to 2030. Antibiotics.

[215] European Food Safety Authority, European Centre for Disease Prevention and Control European Medicines Agency Third Joint Inter-Agency Report on Integrated Analysis of Consumption of Antimicrobial Agents and Occurrence of Antimicrobial Resistance in Bacteria from Humans and Food-Producing Animals in the EU/EEA, JIACRA III. https://www.ecdc.europa.eu/sites/default/files/documents/JIACRA-III-Antimicrobial-Consumption-and-Resistance-in-Bacteria-from-Humans-and-Animals.pdf.

[216] European Medicines Agency Sales of Veterinary Antimicrobial Agents in 31 European Countries in 2019 and 2020. https://www.ema.europa.eu/en/documents/report/sales-veterinary-antimicrobial-agents-31-european-countries-2019-2020-trends-2010-2020-eleventh_en.pdf.

[217] Food and Drug Administration Summary Report on Antimicrobials Sold or Distributed for Use in Food-Producing Animals. https://www.fda.gov/media/154820/download.

[218] Kenyon C. (2022). Positive Association between the Use of Macrolides in Food-Producing Animals and Pneumococcal Macrolide Resistance: A Global Ecological Analysis. Int. J. Infect. Dis..

[219] Tang K.L., Caffrey N.P., Nóbrega D.B., Cork S.C., Ronksley P.E., Barkema H.W., Polachek A.J., Ganshorn H., Sharma N., Kellner J.D. (2017). Restricting the Use of Antibiotics in Food-Producing Animals and Its Associations with Antibiotic Resistance in Food-Producing Animals and Human Beings: A Systematic Review and Meta-Analysis. Lancet Planet Health.

